# Conjugation of VEGFR1/R2-targeting peptide with gold nanoparticles to enhance antiangiogenic and antitumoral activity

**DOI:** 10.1186/s12951-021-01198-4

**Published:** 2022-01-04

**Authors:** Pegah Zanjanchi, S. Mohsen Asghari, Hassan Mohabatkar, Mostafa Shourian, Mehdi Shafiee Ardestani

**Affiliations:** 1 Department of Biotechnology, Faculty of Biological Science and Technology, University of Isfahan, Isfahan, 8174673441 Iran; 2 Institute of Biochemistry and Biophysics (IBB), University of Tehran, Tehran, 1417614411 Iran; 3 Department of Biology, Faculty of Sciences, University of Guilan, Rasht, 4199613776 Iran; 4 Department of Radiopharmacy, Faculty of Pharmacy, Tehran University of Medical Sciences, Tehran, Iran

**Keywords:** Gold nanoparticles, Peptide, VEGFR1, VEGFR2, Tumor growth, Signaling pathways

## Abstract

**Background:**

Inhibition of tumor angiogenesis through simultaneous targeting of vascular endothelial growth factor receptor (VEGFR)-1 and -2 is highly efficacious. An antagonist peptide of VEGFA/VEGFB, referred to as VGB3, can recognize and neutralize both VEGFR1 and VEGFR2 on the endothelial and tumoral cells, thereby inhibits angiogenesis and tumor growth. However, improved efficacy and extending injection intervals is required for its clinical translation. Given that gold nanoparticles (GNPs) can enhance the efficacy of biotherapeutics, we conjugated VGB3 to GNPs to enhance its efficacy and extends the intervals between treatments without adverse effects.

**Results:**

GNP–VGB3 bound to VEGFR1 and VEGFR2 in human umbilical vein endothelial (HUVE) and 4T1 mammary carcinoma cells. GNP–VGB3 induced cell cycle arrest, ROS overproduction and apoptosis and inhibited proliferation and migration of endothelial and tumor cells more effectively than unconjugated VGB3 or GNP. In a murine 4T1 mammary carcinoma tumor model, GNP–VGB3 more strongly than VGB3 and GNP inhibited tumor growth and metastasis, and increased animal survival without causing weight loss. The superior antitumor effects were associated with durable targeting of VEGFR1 and VEGFR2, thereby inhibiting signaling pathways of proliferation, migration, differentiation, epithelial-to-mesenchymal transition, and survival in tumor tissues. MicroCT imaging and inductively coupled plasma mass spectrometry showed that GNP–VGB3 specifically target tumors and exhibit greater accumulation within tumors than the free GNPs.

**Conclusion:**

Conjugation to GNPs not only improved the efficacy of VGB3 peptide but also extended the intervals between treatments without adverse effects. These results suggest that GNP–VGB3 is a promising candidate for clinical translation.

**Graphical Abstract:**

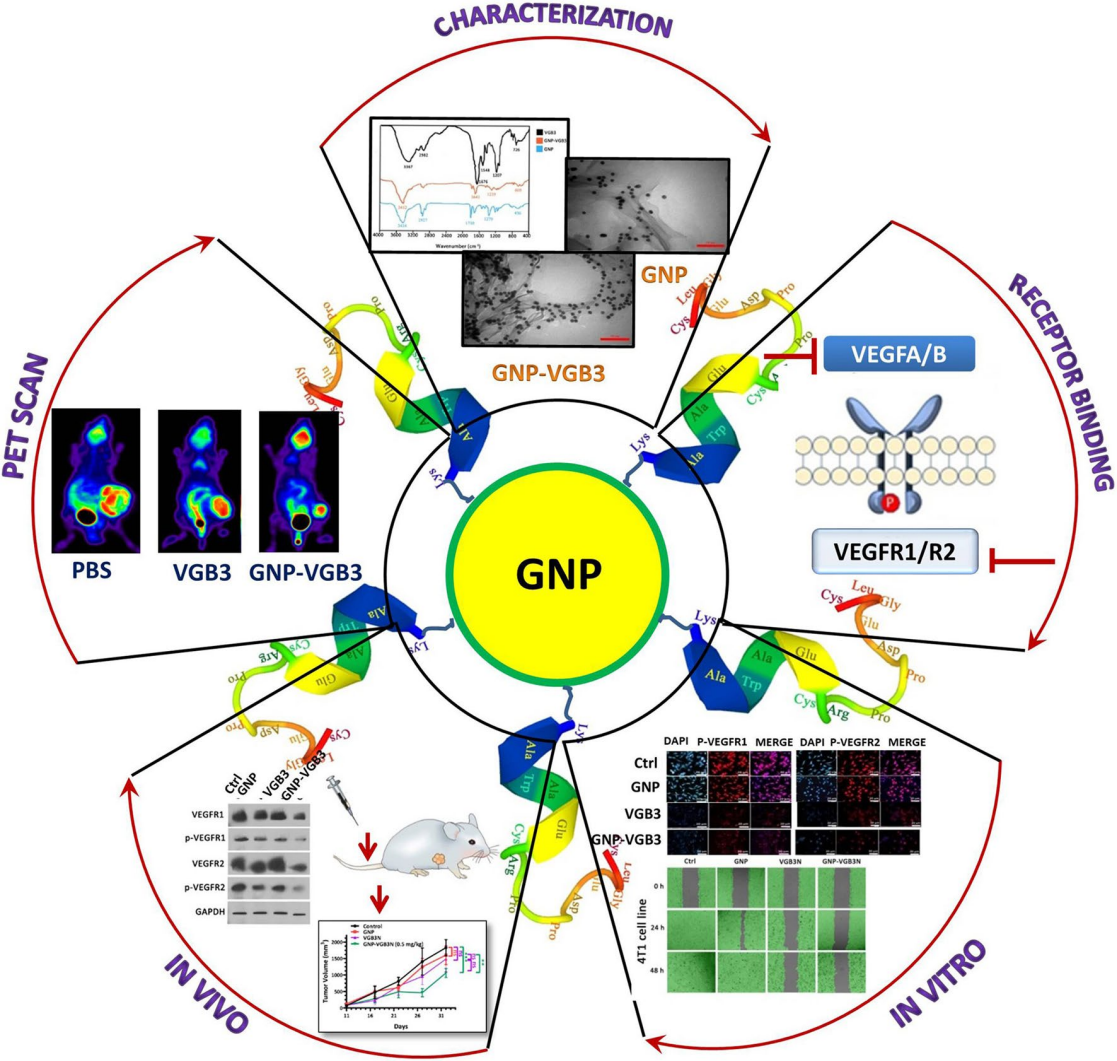

**Supplementary Information:**

The online version contains supplementary material available at 10.1186/s12951-021-01198-4.

## Background

Angiogenesis, the formation and maintenance of blood vessel structures, is critical for providing oxygen and nutrients to tumors during cancer progression and metastasis. Different types of signaling molecules so far have identified as inducers of tumor angiogenesis, among which the vascular endothelial growth factor-A (VEGF-A), also referred to as VEGF, and its receptor VEGF receptor-2 (VEGFR-2) are major activators of tumor angiogenesis [1, 2]. Activation of endothelial cells by VEGF/VGEFR-2 system is dominantly mediated by MAPK/ERK signaling pathway, whereas it less efficiently promotes PI3K/AKT/mTOR signaling pathway [3]. Besides, binding of VEGF-B to VEGFR-1 plays important roles in tumor angiogenesis and metastasis mainly through promotion of PI3K/AKT/mTOR signaling pathway [4]. The experimental findings revealed that the inhibition of tumor angiogenesis is enhanced by simultaneous blockade of both VEGF-A/VEGFR2 and VEGF-B/VEGFR1 cascades [5–9]. So far, however, the common approach for antiangiogenic therapy has been based on the blockade of VEGF/VEGFR-2 signaling pathway [10]. Several types of antiangiogenic drugs have been developed including antibodies (such as bevacizumab), proteins (such as aflibercept and Endostar) and tyrosine kinase inhibitors (TKIs; such as Sunitinib and Sorafenib). However, clinical usage of these drugs is still limited by several factors such as adverse effects, toxicity, acquired drug resistance, and non-availability of valid biomarkers [11, 12]. Peptides have emerged as new generation of therapeutics, as they combine the advantages of small molecules, such as stability and bioavailability, with those of proteins, such as high specificity and potency.

In earlier works, we reported a peptide variant, referred to as VGB3 that binds to both VEGFR1 and VEGFR2, thereby inhibits VEGF-driven proliferation, migration and tube formation of endothelial cells, and tumor growth and metastasis in murine 4T1 mammary carcinoma tumor model [13, 14]. Notably, the observed antitumor effects of this peptide were also associated with the direct influence on 4T1 mammary carcinoma cells, which express both VEGFR1 and VEGFR2. However, peptides have an inherent drawback, i.e., a short serum half-life, which is commonly due to fast renal filtration and enzymatic degradation during systemic circulation. Thus, improved potency and extending injection intervals may be required for the clinical translation of peptides.

Gold nanoparticles (GNPs) are good candidates for therapeutic purposes because they are easy to synthesize and characterize, biocompatible, and binds strongly to functionalities such as amines and thiols [15, 16]. Peptides represent distinctive ligands with different functionalities for binding to GNPs [17]. Moreover, GNPs were found to increase the effectiveness of peptide and protein therapeutics [18]. Therefore, binding to GNPs is a logical choice to increase the potency, half-life and bioavailability of peptide therapeutics.

**Fig. 1:**
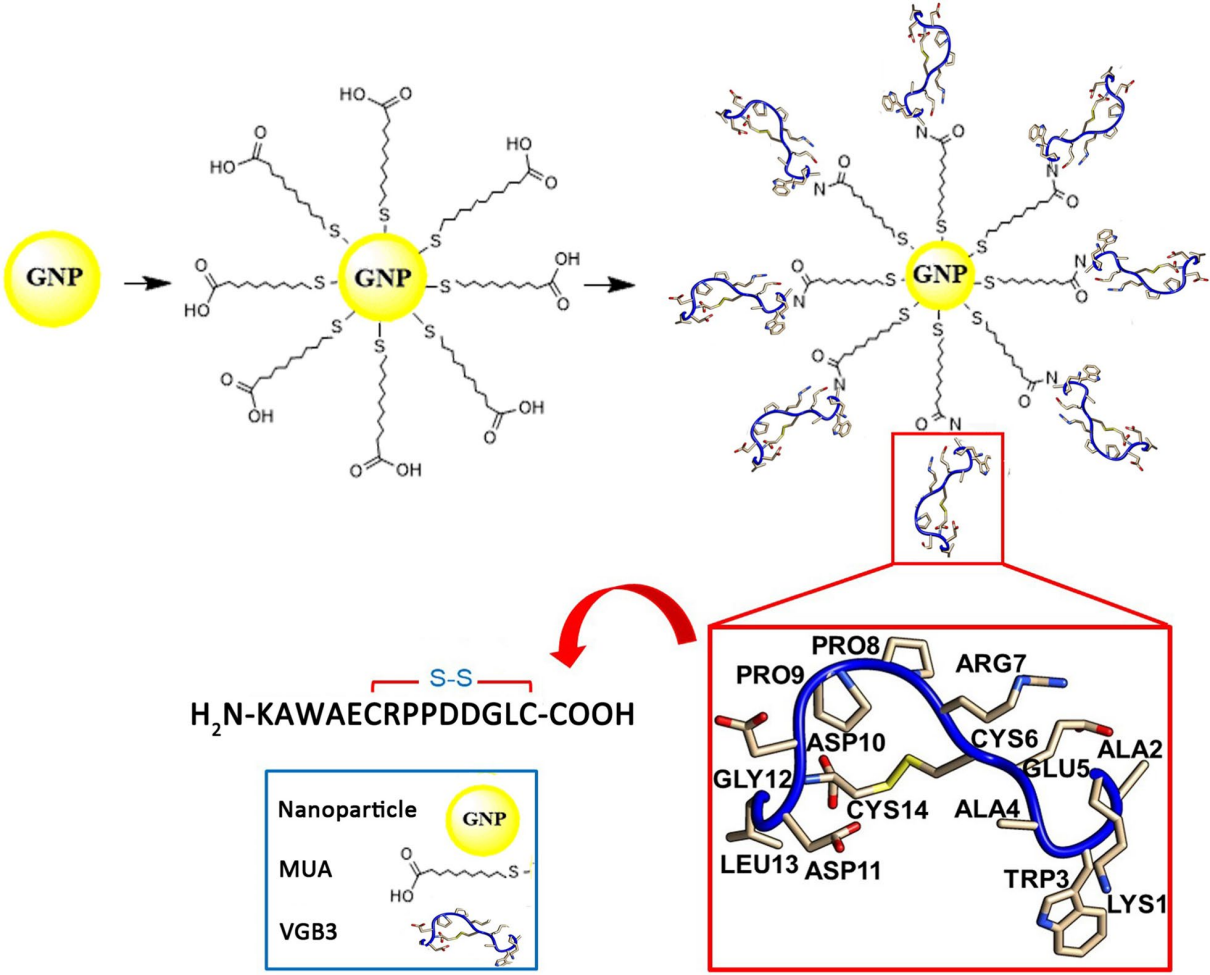
Schematic illustration of VGB3-decorated gold nanoparticles. The particles are coated first with MU/MUA and then coupled to the VGB3 peptide via its linker segment (Lys-Ala-Trp-Ala) to avoid steric hindrance. A three-dimensional model of VGB3 was constructed using homology modeling in MODELLER version 9.18, and the model structure was visualized using UCSF chimera software

Herein, to expand our previous work, VGB3-decorated GNPs are employed for enhanced inhibition of angiogenesis, tumor growth and metastasis (Fig. 1). Detailed in vitro and in vivo studies revealed that the peptide-decorated GNPs can inhibit endothelial and tumor cell functions, and tumor angiogenesis, and enhance the antitumor effect without the treatment-associated adverse effects (Fig. 1).

## Materials and methods

### Materials

All of chemicals, including tetrachloroauric acid (HAuCl_4_), trisodium citrate (Na_3_C_6_H_5_O_7_), absolute ethanol (EtOH), 11-mercapto undecanoic acid (MUA), mercapto undecanol (MU), Tween 20, 2-(*N*-morpholino) ethanesulfonic acid (MES, C_6_H_13_NO_4_S), *N*-(3-dimethylaminopropyl)-*N*′-ethylcarbodiimide hydrochloride (EDC, C_8_H_17_N_3_·HCl), *N*-hydroxysulfosuccinimide (NHS) and bovine serum albumin (BSA) were provided from Merck (Darmstadt, Germany) or Sigma-Aldrich (Milwaukee, WI, USA). Also, deionized double distilled water was used for sample preparation. Phosphate buffer was prepared using appropriate amounts of Na_2_HPO_4_·7H_2_O and NaH_2_PO_4_·H_2_O salts and HCl or NaOH solutions to the desired pH. Bax (sc-7480), Bcl2 (sc-492), Cdk4 (sc-23896), Cyclin D1 (sc-8396), E-cadherin (sc-21791), FAK (sc-271126), p-FAK (sc-81493), VEGFR2 (sc-6251), VEGFR1 (sc-271789), GSK 3B (sc-81462), MMP9 (sc-393859), e-NOS (sc-376751), P53 (sc-126), Paxilin (sc-365379), p-paxilin (sc-365020), p-NFκB (sc-166748), Akt (sc-5298), p-Akt (sc-271966), ERK (sc-292838), p-ERK (sc-16981), GAPDH (sc-32233), p38 (sc-538), p-P38 (sc-17852), PI 3-kinase p85α (B-9) (sc-1637), mTOR (sc-517464), p-mTOR (sc-293133), Raf (sc-7267) and mouse anti-rabbit IgG-HRP (sc-2357), mouse anti-rabbit IgG-FITC (sc-2359) and mouse anti-rabbit IgG-PE (sc-3753) were obtained from Santa Cruz Biotechnology INC, California, USA. Anti-MEK1 + MEK2 antibody [EPR16667] (ab178876), p-VEGFR1 (phospho Y1048, ab192802), p-VEGFR2 (phospho Y1054 + Y1059, ab5473), anti-NF-κB p65 antibody (ab16502) and Rabbit Specific HRP/DAB (ABC) (ab64261), anti-CD31 (Ab32457), anti-Ki-67 (Ab15580) were purchased from Abcam, Cambridge, Uk. N-cadherin (E-AB-70061) and CD31 (E-AB-60608) were obtained from Elabscience Biotechnology Inc., USA. Vimentin (14-9897-82) was used from Thermo Fisher Scientific, San Diego, USA. TUNEL assays were performed using an in situ Cell Death Detection Kit POD (Roche Diagnostic GmbH, Germany).

### Peptide synthesis

The 14 mer peptide with the sequence of 2HN-KAWAECRPPDEGLC-COOH (referred to as VGB3) was synthesized by Shine Gene Molecular Biotech, Inc., (Shanghai, China). The peptide was purified as 90% by high-performance liquid chromatography (HPLC) (Additional file 1: Fig. S1a). The molecular structure of the peptide and the disulfide bond formation was confirmed by electrospray ionization-mass spectrometry (ESI-MS) (Additional file 1: Fig. S1b).

### Preparation of GNP and its conjugation by the VGB3 peptide

Suspensions of gold nanoparticles (GNPs) were prepared by the reduction of tetrachloroauric acid (HAuCl_4_) with trisodium citrate (Na_3_C_6_H_5_O_7_) as described previously [19, 20]. For surface modification of GNPs, MU/MUA solution (8:1 in 2:1 H_2_O/EtOH) was added to the incubated GNPs mixture containing phosphate buffer (PBS, 20 mM, pH8) and Tween 20 (0.2 mg L^−1^). After shaking for 12 h at ambient temperature, the mixture was washed three times by centrifugation at 17,123*g* (30 min) to separate MU/MUA-GNP conjugates. To activate the carboxyl groups of MU/MUA, coated nanoparticles were suspended in MES buffer (0.01 M, pH = 5.5) containing EDC (0.01 M) and NHS (0.02 M) and shaken for 20 min. Then the GNP conjugates were centrifuged at 17,123*g* (30 min) and the precipitate was washed with PBS (0.02 M, pH = 7.4) three times. Subsequently, the solution of GNP-MU/MUA-VGB3 was prepared by the addition of the VGB3 peptide (2 mg peptide dissolved in 96 μL of PBS) to the coated GNPs (4904 μL). After 24 h, the GNPs-peptide was refined from the free peptides by centrifugation at 4 °C (17,123*g* for 30 min). Then, the purified solution was stored at 4 °C for further studies [20].

### Characterization of GNP and GNP-peptide

Different properties of the synthesized NPs including size, shape, superficial charge, and elemental analysis were studied. Therefore, various methods were carried out to assess this information:

(1) For estimating the average size of the synthesized GNPs and GNP-peptide and to determine their content in solution, WPA Biowave II UV–Vis spectrophotometer was used based on the relation between the position of the surface plasmon resonance (SPR) peak and the particle diameters of GNPs [21]. (2) To determine the hydrodynamic radius, size distribution profile in suspension, and surface charge of the synthesized GNPs and GNP-peptide, the dynamic light scattering (DLS) and zeta potential measurements were done using a Zetasizer Ver. 7.11 (Malvern instruments Ltd., UK) [22]. (3) Flame atomic absorption spectrometry (FAAS) (Varian, model AA240FS, USA) and inductively coupled plasma-mass spectrometry (ICP-MS) (Agilent, model 7900 ICP-MS) were used for determination of Au concentration in GNPs and GNP–VGB3 [23, 24]. (4) Fourier transform infrared spectroscopy (FT-IR) (Jasco FT-IR-4700) was employed to confirm the binding of functional groups of MU/MUA on the surface of the synthesized GNPs and peptide. At first, GNPs and GNP-peptide were lyophilized to form powder to mix with spectroscopic grade IR inactive KBr and then pressed in KBr-pellet [23, 24]. (5) The measurements of surface characteristics and the topography of GNP and GNPs-peptide were performed by atomic force microscopy (AFM) (Bruker, model ICON, USA) via spreading the liquid samples onto the mica surface and then heating them [25]. Three and two-dimensional topography of the samples were imaged in the range of 500 nm to 5 μm in order to provide various parameters of surface measurements. (6) Field emission scanning electron microscopy (FESEM) (Zeiss Sigma VP FEI FESEM operated at 10.00 kv) and Transmission electron microscopy (TEM) (Philips CM-10, Netherlands) were used to provide information about the size, shape, morphology and composition of GNPs and GNPs-peptide. Moreover, energy dispersive X-ray spectroscopy (EDS) analysis was performed using FESEM instrument equipped to EDS detector to obtain the elemental composition of synthesized GNPs. In addition, mapping analysis was performed over areas of our samples to trace the dispersion of elements in the surface of GNP-peptide [26].

### Cell culture

The primary normal cells, including Human Umbilical Vein Endothelial cell (HUVEC; C554) and 4T1 mammary carcinoma cell lines (breast cancer cell lines; C604) were obtained from the National Cell Bank, Pasteur Institute of Iran. Both HUVECs and 4T1 cells were grown in Roswell Park Memorial Institute media (RPMI-1640) which consisted of 10% FBS (fetal bovine serum) and 1% penicillin–streptomycin in a moist incubator with 5% CO_2_ at 37 °C until the cells became 90% confluent.

### Binding assay

To evaluate the competitiveness of GNPs, VGB3 and GNP–VGB3 in binding to VEGF receptors, HUVECs were seeded in 96-well plate (1 × 10^4^ cells per well) in DMEM medium with 5% FBS, and then incubated at 37 °C for 24 h. After that time, the cells were transferred to a new culture DMEM medium from which FBS was removed and treated (except the control) with different concentration of free VGB3 (500, 700 and 1200 ng mL^−1^), GNP–VGB3 (250, 500, 1000 ng mL^−1^) and free GNPs in the presence of VEGF (20 ng mL^−1^) at 37 °C overnight. To fix the cells, paraformaldehyde 4% (in PBS) solution was used for 10 min at room temperature, followed by washing three times with ice-cold PBS. Next, for permeabilization, the samples were incubated with PBS + 0.1 − 0.25% Triton X-100 for 10 min. After three washes with PBS, the cells were incubated with 1% BSA + 22.52 mg mL^−1^ glycine in PBST (PBS + 0.1% Tween 20) for 30 min at room temperature to block unspecific binding of the antibodies. The cells were incubated with primary anti-VEGFR1 and anti VEGFR2 antibodies together for 1 h at room temperature and after washing thrice with PBS, the cells were incubated with Fluorescein isothiocyanate (FITC)-labeled rabbit anti-mouse IgG secondary antibody for 1 h at 37 °C in the dark position. 4′,6-diamidino-2-phenylindole (DAPI) (0.1–1 μg mL^−1^) was used as a counterstaining for 1 min and after rinsing with PBS, the cells were mounted and observed under a fluorescence microscope which the cells were visible in blue by FITC labeling.

All steps were performed as the same as described above for the binding assay of free GNPs, free VGB3, and GNP–VGB3 to phospho-VEGFRs with different labeling. Anti-p-VEGFR1 antibody and anti-p-VEGFR2 as a primary antibodies were detected with phycoerythrin (PE) labeled goat anti-mouse IgG secondary antibody which made the cells recognizable as red in the images obtained by fluorescent microscope.

### Cell viability assay

The cytotoxicity of GNPs, VGB3, and GNP–VGB3 was evaluated by MTT (3-[4, 5-dimethyl-2-thiazolyl]-2, 5 diphenyl tetrazolium bromide) method on HUVEC and 4T1 cells after 24 and 48 h in the presence and absence of VEGF (20 ng mL^−1^). First, HUVEC and 4T1 cells were seeded around 1 × 10^4^ into 96-well plates in RPMI media comprising 10% FBS and 1% penicillin–streptomycin and incubated with 5% CO_2_ overnight at 37 °C. After that, the various concentrations of free GNPs, VGB3, and GNP–VGB3 (5–2000 ng mL^−1^) were used for the treatment of the cells to be compared with the untreated cells in the presence of VEGF (200 ng mL^−1^) for 24 h. Then, 150 μL of fresh culture medium and 50 μL MTT solution (2 mg mL^−1^ PBS) were substituted with the previous media in each well and incubated at 37 °C for 4 h. After the incubation, the medium was replaced with 200 μL of DMSO to dissolve the insoluble purple formazan in viable cells by its mitochondrial enzymes. Subsequently, the observance of each plate was evaluated at the wavelength of 570 nm by Eliza reader (Sunraise, TCAN Co., Austria).

### Scratch healing assay

The scratch healing assay was utilized to measure the in vitro cell migration rate. At first, HUVE and 4T1 cells were cultured into a 12-well plate that seeded 1 × 10^4^ cells per well until cells became confluent to form a monolayer. To create a linear wound after 24 h, the monolayer was scratched using 200 μL sterile plastic pipette tip and the cells were washed with PBS. Afterward, the cells were treated with IC_50_ concentration of free GNPs (335,447 ng mL^−1^), VGB3 (710 ng mL^−1^), and GNP–VGB3 (554 ng mL^−1^) in the absence and presence of VEGF (20 ng mL^−1^) and untreated cells were considered as a control group and then incubated for 24 h. Thereafter, the cells were washed with PBS twice and the migration percentage of each group of migrated cells at time 0 (T_0_) was examined via an inverted microscopic (LABOMED, TCM400, USA) at 4× magnification according to the wound distance. By maintaining the previous conditions, this assay was performed two more times at 24 and 48 h (T_24_ and T_48_).

### Detection of apoptosis using flow cytometry

HUVECs and 4T1 cells were seeded at a density of 1 × 10^4^ cells per well in the RPMI 1640 media culture with 10% PBS to determine and measure the apoptosis of cells affected by free GNPs, free VGB3, GNP–VGB3, after 24 h of incubation, the media were replaced with 2 mL fresh ones involving IC_50_ concentration of every treatment, and the incubation was continued for 48 h. Thereafter, the cells were separated by trypsinization and 1× binding buffer was added to the cell sediment obtained from the centrifugation. Afterward the cells were stained via annexin V-FITC/propidium iodide (PI) apoptosis detection kit (Exbio, Czech Republic) based on the manufacturer’s protocol, and then incubated at room temperature for 15 min. Finally, Flow cytometry (MACS Quant 10; Miltenyi Biotech GmbH) was used to analyze the annexin V-FITC/PI binding to the diagnostic data about apoptotic and necrotic cells which were accessible by using the FlowJo software package (Treestar, Inc., San Carlos, CA) [27].

### Measurement of intracellular ROS levels

The ROS assay is a cell-based experiment allocated to evaluate Reactive Oxygen Species (ROS) activity within a cell by the cell-permeable fluorogenic substrate, 2ʹ, 7ʹ-dichlorodihydrofluorescein diacetate (DCFDA) via flow cytometry [28]. Briefly, HUVECs and 4T1 cells were seeded at 1 × 10^4^ cells per well in 96-well plates and incubated at 37 °C for 24 h. After washing the cells with sterile PBS, the cells were treated with free GNPs, free VGB3, and GNP–VGB3 with their IC_50_ concentration before excitation of oxidative stress in the absence and presence of VEGF (20 ng mL^−1^). The cells were stained with 10 μM L^−1^ of DCFDA for 1 h which this dye was used to check the ROS generation in intact cells. After that, the cells were analyzed by the flow cytometer by reading the signals at Ex/Em: 485/535 nm to measure the fluorescence intensity arising from the ROS changes.

### Cell cycle assay

To evaluate the cell cycle, the cells were stained with propidium iodide (PI) and then analyzed by flow cytometry [29]. Firstly, HUVE and 4T1 cells were cultured with trypsin 24 h after treatment with free GNPs, free VGB3, and GNP–VGB3 that 1 × 10^4^ cells per well were incubated in 6-well plates. After incubation for 2 days, the cells were fixed in ethanol (70%) in 4 °C (30 min) for more than 2 h and then centrifuge the ethanol-suspended cells to decant ethanol thoroughly. Finally, cells after washing with PBS, the cells were stained with 1 mL DAPI/Triton X-100 staining solution and keep 30 min in the dark. The excitation of DAPI that requires a UV light source is not generally available, but the emission of DAPI is measured in the blue wavelengths. The synthesized DNA and the qualification of the cell cycle were analyzed by flow cytometry (MACS Quant 10; Miltenyi Biotech GmbH) to assess stages of the cell cycle according to a cell count Vs DAPI plot.

### Antitumor activity of treatments in tumor-bearing mice

Thirty female BALB/c mice (4–6 weeks) were prepared from the Laboratory Animal Center of the Iran Pasteur Institute and supported according to the Tehran University of Medical Sciences’ Institutional Animal Care and Use Committee (IACUC). Healthy mice were maintained under the animal ethics, proven conditions with free access to sterile food and water in 12 h light–dark phases. To generate 4T1 tumor models, tumor cells (4T1; 1 × 10^6^ cells/500 μL or 1 × 10^5^ cells/50 μL) were injected hypodermically into the right flanks of 3–5 mice. After that, the 4T1 tumors were removed from the body of the breast cancer mice and then divided into small pieces (under 0.3 cm^3^), and transplanted into the BALB/c’s right flanks under ketamine (100 mg kg^−1^, i.p.) and xylazine (10 mg kg^−1^, i.p.) anesthesia. When the size of the tumors was reached to 200 mm^3^, the cancerous mice were randomly divided into five groups of six mice. Next, for evaluation, the antitumor efficacy of GNPs, VGB3, and GNP–VGB3, mice were injected intravenously with a dose of 0.5 mg/kg (in 100 μl) of GNP–VGB3, a dose of 0.5 mg/kg (in 100 μl) of VGB3 and 8.9 mg kg^−1^ of GNPs through their tail vein weekly, whereas the control group just received sterile PBS. By measuring the length and width of the tumors every 5 days via a digital Vernier caliper (Mitutoyo, Japan), the tumor volume was calculated by using the following formula: Volume = length × width^2^ × 0.52 [30]. In addition the survival percent and the body weight of these tumorous mice were measured every 5 days.

To produce three-dimentional color images to detect the signs of cancer and to assess the severity of its progression in the body, positron emission tomography (PET) scan is used. The diseased cells are diagnosed by an injectable radioactive detector. Firstly, each of the four groups of Balb/c mice at the end of treatments (day 32) fasted for 8 h were anesthetized by anesthesia drugs and then 14.8 MBq 2-deoxy-2-[^18^F] Fluoro-d-glucose (FDG) were injected via the tail vein. 24 h after the radioactive tracer injection, each mouse was placed on the animal bed for imaging with the clinical PET/CT scanner to obtain the amount of radiopharmaceutical absorption of the tumor tissue in the body.

### MicroCT imaging

For in vivo CT imaging, GNP–VGB3 and GNP (150 μL, Au = 2.97 mg L^−1^) were injected into mice via the tail vein and one group considered as a blocking group which received VGB3 (0.5 mg kg^−1^) at first and after 1 h, GNP–VGB3 was injected. All groups were compared with untreated group considered as a control group which only received PBS. The mice (n = 3) were then anesthetized and subjected to CT imaging using a CT scanning system at 3 h post-injection. The Au element of samples as a contrast agent of CT imaging was investigated. So, for analysis of biodistribution, an in vivo X-ray Micro-Computed Tomography (micro-CT) scanner (LOTUS inVivo, Behin Negareh Co., Tehran, Iran) was exploited at the Preclinical Core Facility (TPCF) based at Tehran University of Medical Sciences. LOTUS-inVivo has a cone beam micro-focus X-ray source and a flat panel detector. To obtain the best possible image quality, the X-ray tube voltage and its current were set to 50 kV and 120 µA, respectively, and frame exposure time set to 2 s by 1.7 magnifications. Total scan duration was 49 min. Slice thicknesses of reconstructed images were set to 30 µm. All the protocol setting process was controlled by LOTUS-inVivo-ACQ software. The acquired 3D data was reconstructed using LOTUS inVivo-REC by a standard Feldkamp, Davis, Kress (FDK) algorithm.

### ICP-MS analysis of isolated tissues for quantification of Au element

The in vivo biodistribution of GNP–VGB3, VGB3 and GNPs in major organs, including liver, spleen, kidney, tumor and heart was investigated by inductively coupled plasma-mass spectrometry (ICP-MS, Agilent 7500, America) after 24 h post-injection on 4T1-bearing mice to determine Au concentration. So in brief after micro CT imaging, the organs were cut immediately and a small pieces of them were digested in 5 cc of HNO3 for 24 h to dissolve the Au nanoparticles. The collected tissue samples were then incubated at 100 °C until the samples were completely dissolved in acid. The mineralized samples were diluted in deionized water to reach a volume of 15 cc to be prepared for elemental analysis using ICP-MS. For each sample, the concentration of Au element was evaluated and determined based on percentage of injection dose per tissue (%ID/tissue).

### Immunohistochemistry staining

For immunohistochemical (IHC) analysis, the tumor tissues were excised from the treated and untreated mice and then fixed in formalin (4%), prepared in paraffin sections, and de-paraffinized and hydrated by xylene and ethanol, respectively. Thin tissue segments were stained with hematoxylin and eosin (H&E). Then, the stained sections were incubated with the primary mouse monoclonal antibodies for CD31 (as IHC markers of endothelial cells and vascular differentiation), Ki67 (as a marker for cell proliferation) and TUNEL (to assign apoptotic induction) at 4 °C for overnight. Biotinylated Goat Anti-Polyvalent was used as a secondary antibody to cover all tissue sections to localize the specific antibodies and this level was followed by the addition of a streptavidin-enzyme conjugate to bind to the biotin on the secondary antibody. Next, 3,3-diaminobenzidine (DAB) was added to tissue sections to detect the antigens, and after that hematoxylin was used as a counterstain. Finally, the images were obtained by the microscope.

### Western blot analysis

Isolated 4T1 tumor tissues from control and treated groups with free GNPs, free VGB3s, and GNP–VGB3 were lysed with lysis buffer containing 500 μL Tris-HCl, pH 8.0, 0.003 g EDTA, 0.08 g NaCl, 0.025 g sodium deoxycholate, 0.01 g sodium dodecyl sulfate (SDS), 1 tablet protease inhibitor cocktail and 10 μL Triton X-100 (NP40 (1%)). For assessing the protein concentrations, the Bradford assay was performed. Afterward, the lysed cells were separated according to their size by SDS polyacrylamide gel and then transferred into polyvinyl difluoride (PVDF) or nitrocellulose western blotting membranes. Next, in the blocking level, the blocking solution was used to cover the membrane to prevent the non-specific reaction of primary antibodies. At the end of the membranes blocking time, the membranes were incubated with primary antibodies against GAPDH, Bax, Bcl2, Cdk4, Cyclin D1, E-cadherin, N-cadherin, FAK, p-FAK, VEGFR2, VEGFR1, p-VEGFR1, p-VEGFR2, GSK3B, MMP9, NFKB, p-NFkB, e-NOS, P53, Paxillin, p-paxillin, Akt, p-Akt, ERK, p-ERK, MEK1/2, mTOR, p-mTOR, p38, p-P38, PI3K, Raf and Vimentin (1:300) overnight at 4 °C. After that, the membranes were washed in tris-buffered saline and tween 20 (TBST) for three times and incubated with mouse anti-rabbit IgG conjugated to horseradish peroxidase (HRP) secondary antibody (1:1000) for 1 h and 15 min at room temperature. The detection of protein bands was performed using ECL chemiluminescence reagents and for protein normalization, GAPDH was used as a loading control.

### Statistical analysis

To analyze the obtained data, to draw graphs and for statistical analysis, the Prism software (version 8.0.2 for Windows, GraphPad Software, La Jolla, California, USA; www.graphpad.com) was used. All data were prepared based on mean ± SEM. For assessing the significant differences in multiple comparisons between more than two groups with one independent variable, one-way ANOVA by Turkey’s post hoc test was used, whereas for two independent variables, two-way ANOVA was performed that reported statistical confidence by comparing every mean with every other means. The amount of statistical significance in all analyses was more than 95% confidence level (P value less than 0.05).

## Results and discussion

### Characterization of the synthesized naked and peptide-conjugated GNPs

**Fig. 2:**
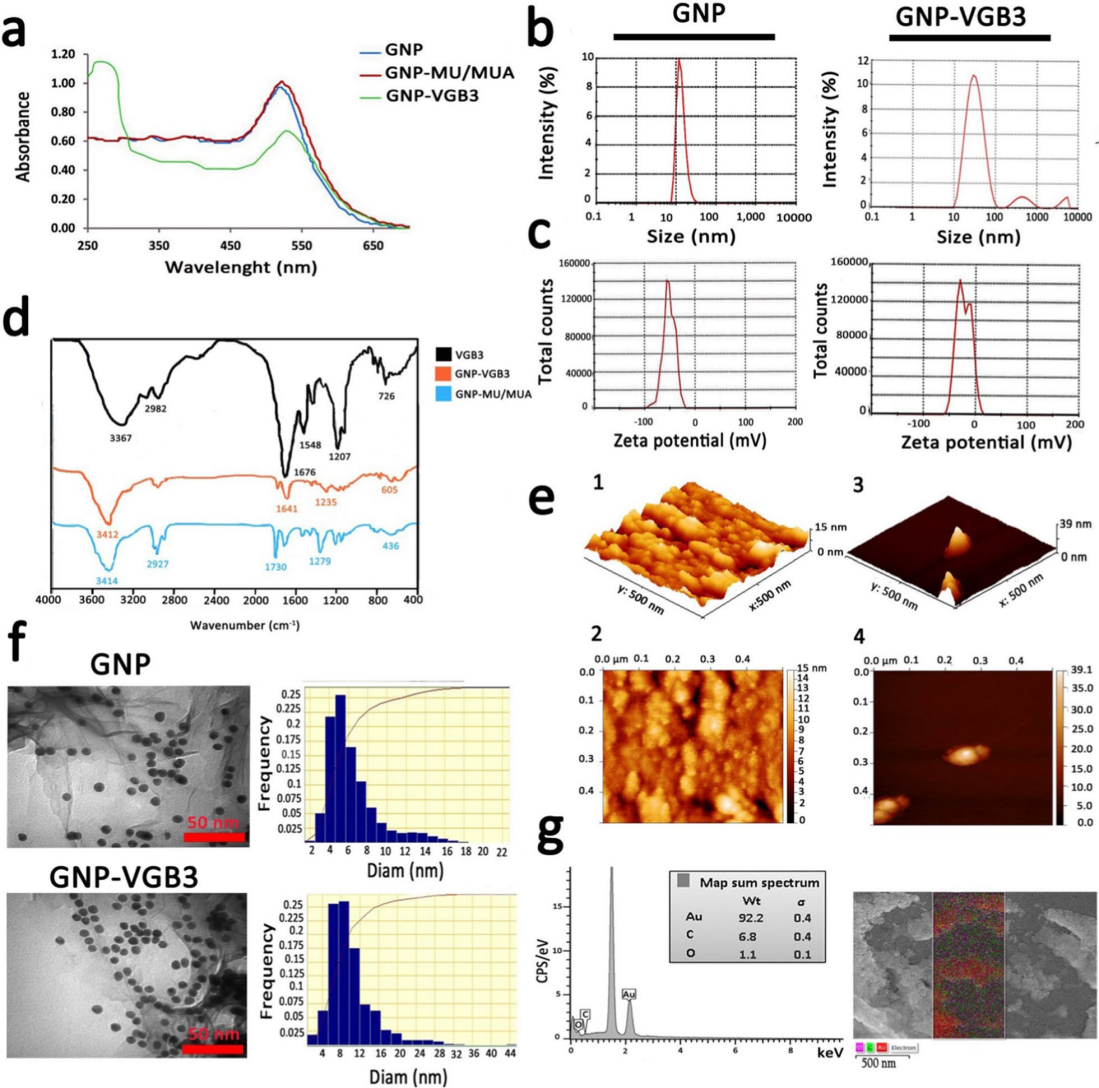
The characterization of the synthesized naked and conjugated GNPs. **a** UV–Vis absorption spectra of GNPs before and after modifications. **b** DLS curves of GNPs and GNP–VGB3 related to hydrodynamic diameters. **c** The zeta potentials of GNPs and GNP–VGB3. **d** FT-IR spectra of GNP and VGB3 and their conjugations in the 400–4000 cm^−1^ region. **e** AFM of GNP and GNP–VGB3. (1, 2) AFM analysis depicted the mean diameter of GNPs in 3D and 2D images, respectively (scale bar: 500 nm), and (3, 4) In the same way, the obtained images from GNP–VGB3 were dedicated within a scan area of 0.5 μm × 0.5 μm. **f** TEM images to verify the size, shape and the size distributions of GNPs and GNP–VGB3, respectively (scale bar: 50 nm) and **g** EDS analysis of GNPs-VGB3 and its mapping to identify each element in the sample (Au: red; C: green, and O: Violet)

GNPs were prepared as described previously [19, 20]. Characterization of GNPs using the UV–Vis spectra (400–700 nm) showed an absorption band shift towards higher wavelengths (red shift), indicating the increase of GNP size after surface modification. The synthesized GNPs showed a strong absorption at λ_max_ ~ 516 nm, which is attributable to globular GNPs with diameters lower than 20 nm [31, 32]. Addition of MU/MUA to the surface of GNPs led to a notable red shift from λ_max_ ~ 516 nm to 522 nm. After conjugation of VGB3 to the modified GNPs, a considerable shift from λ_max_ ~ 522 nm to around ~ 550 nm and a new single absorption band were emerged at λ_max_ ~ 280 nm, which the later can be associated with the tryptophan residue in the structure peptide (Fig. 2a). Based on measurements by UV standard curve, we have detected no unconjugated peptide fraction in the reaction mixture.

According to the DLS graphs (Fig. 2b), the mean diameter of GNPs was 16 nm and the size distribution was between 7–40 nm, whereas the average diameter of GNP-peptide was 30, indicating a slightly more variation than free GNP. The value of Zeta potential, an indicator of dispersion stability and the tendency of GNPs to aggregate in solution, indicated that GNPs were negatively charged, which is due to the citrate ions. Immobilization of the positively-charged peptide molecules onto highly-negatively charged (− 51.4 ± 0.88 mv) GNPs decreased the negative charge to − 23.9 ± 0.55 mv (Fig. 2c).

Next, the FAAS and ICP-MS analysis was performed to achieve the gold concentration. Accordingly, the Au concentration was 48 mg L^−1^ for free GNP and 19 mg L^−1^ for GNP–VGB3 (data not shown).

The FT-IR spectra enable us to address the functional groups in the structure of synthesized peptide, MU/MUA modified GNP, and the peptide immobilized on modified GNPs. As indicated in Fig. 2d, VGB3 was bonded from its NH_2_ group to the –COOH functional groups of MU/MUA modified GNP. In MUA modified GNP, the absorption bands in 3414, 1730 and 1279 cm^−1^ can be related to the stretching vibrations of hydroxyl group of COOH, –C=O and –C–O bands in the MU/MUA linker, respectively. The band at 2927 cm^−1^ was assigned to the stretching vibrations of –C–H in –CH2– groups of MU/MUA. The broad intense absorption band in the wavenumbers of 3367 cm^−1^ (in VGB3 spectrum) and 3412 cm^−1^ (in GNP–VGB3 spectrum) was related to the vibrations of the hydroxyl group of –COOH and –NH groups in the peptide [24]. The stretching vibrations of amidic –C=O band in the peptide were observed at 1676 cm^−1^ (for unconjugated peptide) that overlapped by the vibrations of the carbonyl group of COOH. In both VGB3 and GNP–VGB3 spectra, the stretching vibrations of amidic –NH was occurred in 3150–3350 cm^−1^ that overlapped with –COOH band and the vibrations of –C–S band was appeared in 600–700 cm^−1^. The bands in 1207 and 2982 cm^−1^ can be assigned to the vibrations of –C–N of amid group and –C–H in CH2, respectively. Also, the bending vibrations of NH observed in 1548 cm^−1^ represented the amide bands. In the GNP–VGB3 spectra, reduction in the band intensity of the carbonyl group of COOH in 1730 cm^−1^ and increase in the intensity of the band at 1641 cm^−1^ is related to amidic –C=O vibrations, indicating the formation of amid band by the covalent linkage between the NH_2_ group of peptide with –COOH group of MU/MUA conjugated GNP [23, 33].

Figure 2e(1–4) shows atomic force microscopy (AFM) images of naked GNP and GNP-peptide within a scan area of 0.50 μm × 0.50 μm for both samples. In three dimensional images, the average size of GNPs and GNP-peptide were estimated about 15 ± 4 nm and 39 ± 4 nm, respectively [25].

TEM and FESEM were utilized to specify the morphology of NPs. According to the results obtained from TEM, the shape of free GNPs and GNP-peptide were spherical with a monodispersed size from 5–12 nm (Fig. 2f). The differences in the mean diameters of nanoparticles obtained by TEM and DLS could be due to the methods of size measurement. FESEM images of GNP-peptide is shown in Additional file 1: Fig S2 that displayed surface morphology of this sample. Compared to GNP–VGB3 displaying the mean size of 25 nm, free GNP displayed much larger size, reflecting the susceptibility of free GNPs for aggregation. Based on EDS analysis, the fundamental elements of the nanoparticles were the Au element (92.2%), C and O elements (6.8 and 1.1%, respectively). The presence of C and O elements is attributable to the MU/MUA linkers, and the peptide molecules on the surface of GNPs.

### GNP-VGB3 recognizes and neutralizes VEGFR1 and VEGFR2

**Fig. 3:**
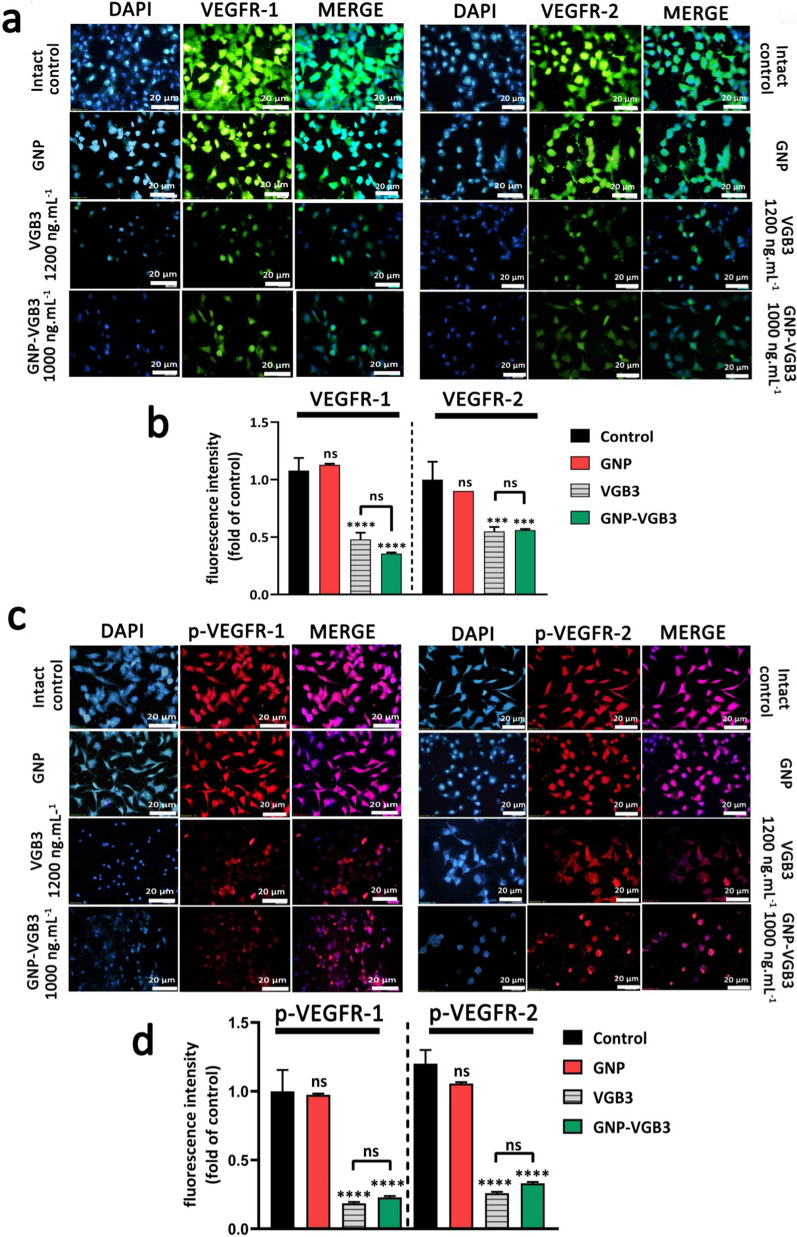
**GNP-VGB3 recognizes VEGFR1 and VEGFR2 and suppresses their VEGF-induced phosphorylation in endothelial cells. a** Immunocytochemical images of HUVE cells treated with PBS, GNP, VGB3 and GNP–VGB3 using FITC-secondary anti-mouse antibody (green) to bind to VEGFR1 (left) and VEGFR2 (right) (scale bar: 20 μm). **b** Statistical analysis of VEGFR1/2 fluorescence intensity under various concentrations were performed by prism software 8; Oneway ANOVA and all data displayed mean ± SEM (n = 3). **c** Immunoflourescent staining images (PE-secondary anti-mouse antibody (red)) of phosphorylated-VGEFR1 (p-VEGFR1) and p-VEGFR2 with various treatments. **d** Quantitative analysis of the fluorescence intensity of p-VEGFR1/2 by prism software 8 analyzed by One-way ANOVA for different treatments. (****P < 0.0001, ***P < 0.001 and NS: not significant in comparison with control)

VEGFR2 and VEGFR1 are highly expressed on the surface of endotheliral cells (ECs) [34]. The specific cell binding of GNP–VGB3 to VEGFR1 and VEGFR2 were investigated by immunocytochemical assay using human umbilical vein endothelial cells (HUVECs). Pre-incubation of HUVECs by increasing concentrations of free VGB3 (500, 700 and 1200 ng mL^−1^) and GNP–VGB3 (250, 500, 1000 ng mL^−1^) reduced binding of fluorescently labeled anti-VEGFR1 or anti-VEGFR2 (20 ng mL^−1^), whereas fluorescence intensities was not affected when HUVECs treated with GNP (Additional file 1: Fig. S3). These results indicate that VGB3 retained its ability to recognize VEGFR1 and VEGFR2 after conjugation to the gold nanoparticles (Fig. 3a, b). In addition, GNP–VGB3 treatment inhibited VEGF-induced phosphorylation of VEGFR2 and VGEFR1. As indicated in Fig. 3c, d, incubation with GNP–VGB3 (1000 ng mL^−1^) as well as VGB3 (1200 ng mL^−1^) reduced fluorescent signals of anti-phospho VEGFR1 (anti-pVEGFR1) or anti-pVEGFR2 in a dose-dependent manner compared to controls, whereas binding of the antibodies was not affected by GNP (Additional file 1: Fig S4). These results indicate that GNP–VGB3 abrogated the VEGF-induced activation (phosphorylation) of VEGFR1 and VEGFR2.

### Inhibition of proliferation and migration of endothelial and tumor cells

**Fig. 4:**
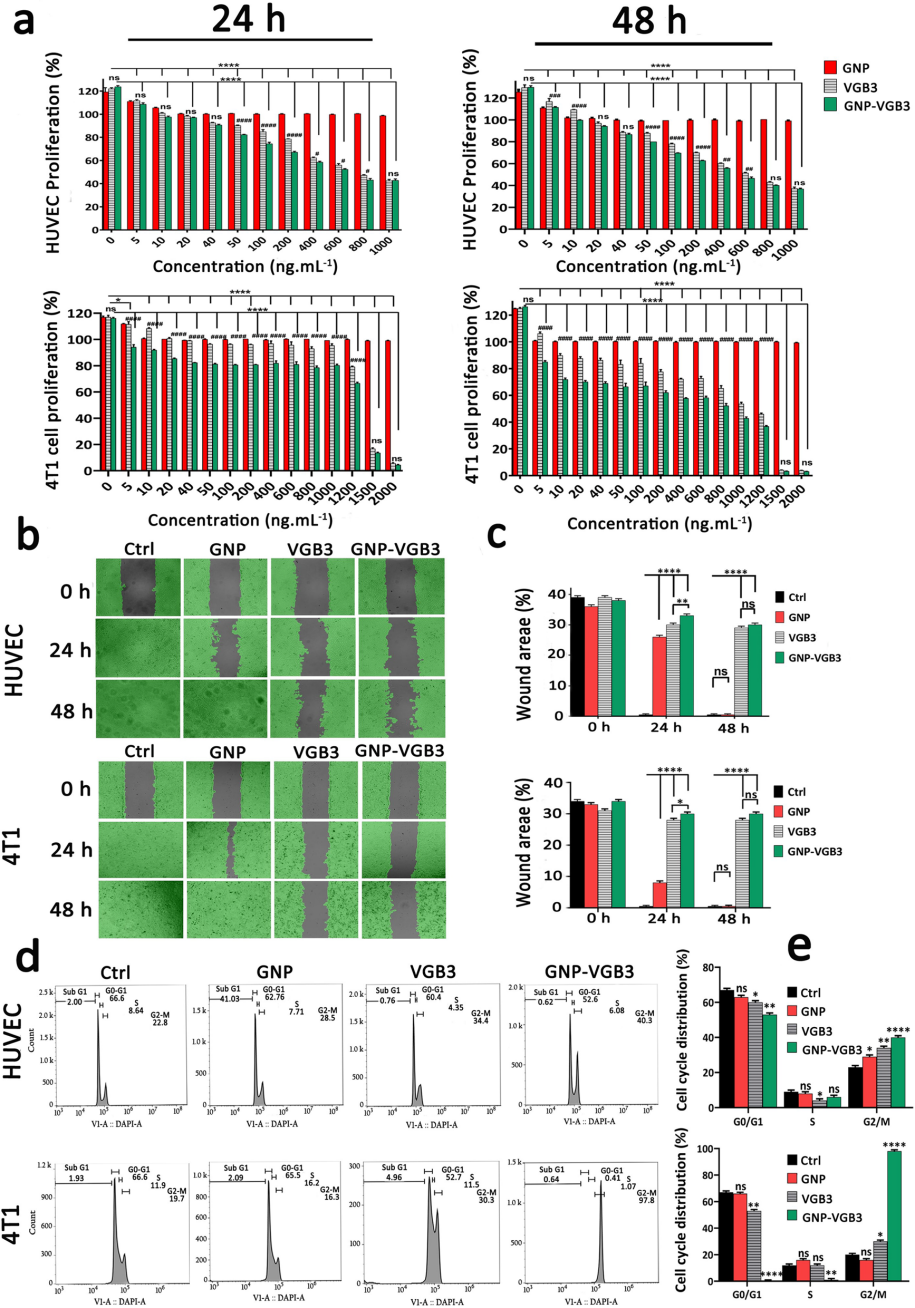
Inhibition of HUVE and 4T1 cells proliferation, migration and cell cycle progression. **a** The investigation of cell viability in the HUVECs and 4T1 cells after treatment with different concentrations (0–1000 ng mL^−1^) of GNPs, VGB3, and GNP–VGB3 in the presence of VEGF (20 ng mL^−1^). Also, the cell viability rates of 4T1 cells after treatments with different concentrations (0–2000 ng mL^−1^) of GNPs, VGB3 and GNP–VGB3 after 24 and 48 h incubation. The cell viability rates of treated cells were evaluated by MTT assay for 24 and 48 h incubation using prism software 8 (One-way ANOVA method, based on mean ± SEM of six independent observations). **b** HUVE and 4T1 cells wound closure cell migration assay after different treatment with PBS, GNPs, VGB3, or GNP–VGB3 in the presence of VEGF (20 ng mL^−1^) after incubation for 0, 24 or 48 h. By Wimasis image analysis, wound areas in the images were shown with a gray color. **c** Statistical analysis of wound area of HUVE and 4T1 cells after treatments by One-way ANOVA, mean ± SD, n = 3). **d** Cell cycle analysis of HUVE and 4T1 cells after exposure to PBS, GNPs, VGB3, and GNP–VGB3 and VI stain followed by flow cytometry analysis. **e** Cell cycle distribution of HUVE and 4T1 treated cells were analyzed statistically by One-way ANOVA pathway (mean ± SEM, and n = 3, ****P < 0.0001, **P < 0.01, *P < 0.05, or ns: not significant)

VEGFR1 and VEGFR2 undergo dimerization and VEGFR ligand-dependent phosphorylation, which trigger mitogenic, chemotactic, and prosurvival signals, along with stimulation of tumor vessel formation [35]. We have previously indicated that VGB3 can inhibit proliferation, migration and tube formation of HUVECs, and proliferation of 4T1 mammary carcinoma tumor cells that expresses both VEGFR1 and VEGFR2 [13]. Here, to confirm whether VGB3 retained its effects in nanoformulation, the antiproliferative and antimigrative properties of GNP–VGB3 were determined in comparison to GNP and VGB3 in HUVE and 4T1 cells when stimulated by VEGF (20 ng mL^−1^). Notably, blank-GNP had no significant effects on the cell viability and showed a similar result to the non-treated cells, inferring that the blank-GNP composition is biocompatible (Fig. 4a). In contrast, VGB3 and GNP–VGB3 exhibited time and dose-dependent cytotoxicity; the half-maximal inhibition (IC_50_) values of GNP–VGB3 against HUVECs were 554 ng mL^−1^ (24 h) and 440 ng mL^−1^ (48 h) and for free VGB3 were 710 ng mL^−1^ (24 h) and 561 ng mL^−1^ (48 h). Similarly, the data of 4T1 cells demonstrate that the IC_50_ values of GNP–VGB3 were 1238 ng mL^−1^ (24 h) and 423 ng mL^−1^ (48 h) and for free VGB3 were 1971 ng mL^−1^ (24 h) and 771 ng mL^−1^ (48 h). These results in agreement with previous studies indicated that the cytotoxicity of peptide-conjugated GNP was higher than that of free peptides [36, 37].

Cell migration is essential for angiogenesis of endothelial cells and invasion of tumor cells. We carried out wound healing assay to investigate the antimigrative effect of GNP–VGB3 in HUVE and 4T1 cells. Based on the above mentioned IC_50_ values, endothelial cells were incubated with VGB3 (710 ng mL^−1^), GNP–VGB3 (554 ng mL^−1^) for 24 and 48 h, and filling the scratch area with cells was evaluated compared to controls and GNP-treated  cells. When stimulated by VEGF (20 ng mL^−1^), HUVE and 4T1 cells were able to fill the wound area after 24 h. However, the VEGF-induced migrations were inhibited by VGB3 and GNP–VGB3, and to a lesser extent by GNP after 24 h and suppression was maximal in GNP–VGB3-treated groups (P < 0.0001). After 48 h, whereas GNP-treated HUVE and 4T1 cells completely migrated to the wound area, VGB3 and GNP–VGB3 potently inhibited VEGF-induced migration of cells compared to controls and GNP-treated cells (P < 0.0001). Results of wound healing assay indicated that GNP–VGB3 could suppress endothelial and tumor cells locomotion in response to growth factor-attractive surroundings (Fig. 4b, c).

The induction of cell cycle arrest is a strategy to control aberrant cancer cell proliferation [38]. Hence, to investigate the mechanism of antiproliferative effects, we evaluated the cell cycle distribution using flow cytometry. Cell cycle analysis of HUVECs and 4T1 cells exposed to GNP, VGB3 and GNP–VGB3 is shown in Fig. 4d. Consistent with the results of proliferation and migration, treatment with VGB3 and GNP–VGB3 resulted in an arrest in the G2/M phase, with a significant decrease in G0/G1 phase versus control cells, whereas GNP was ineffective in 4T1 cells or moderately (P < 0.05) effective in HUVECs. Importantly, the accumulation in G2/M phase was significantly higher in HUVE and 4T1 cells treated GNP–VGB3 (40.3 and 97.8%, respectively) than in cells treated with free peptide (34.4 and 30.3%, respectively)(Fig. 4e), suggesting that binding to GNP induced the inhibitory effects of VGB3. These results are consistent with the data obtained by MTT and scratch analyses and provide further evidence for enhanced potency of VGB3 after as a result of ligation to GNPs.

### Induction of ROS production and apoptosis in endothelial and tumor cells

**Fig. 5:**
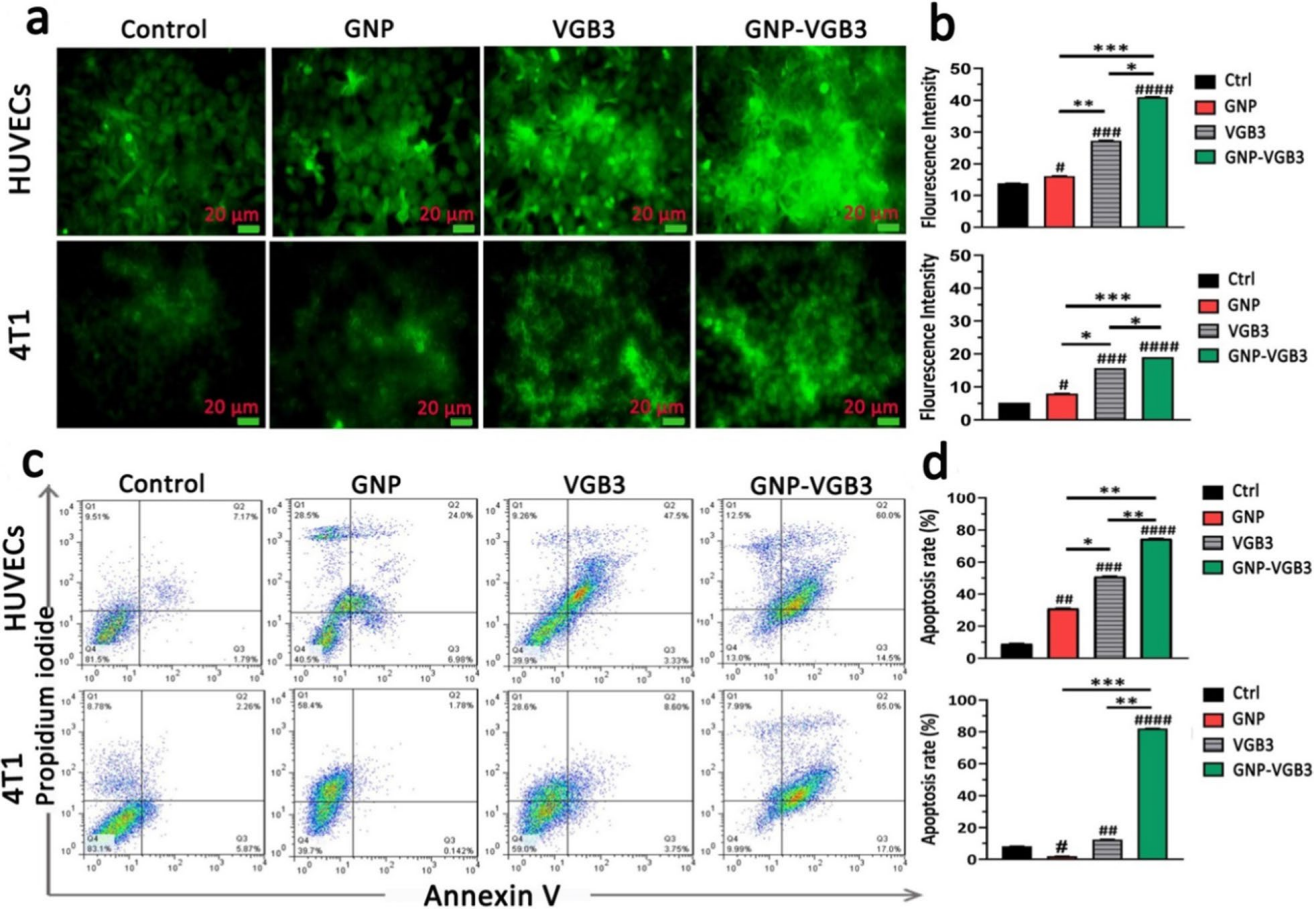
ROS overproduction and apoptosis induction in HUVE and 4T1 cells. **a** Intracellular ROS induction of HUVE and 4T1 cells treated with PBS, GNPs, VGB3 and GNP–VGB3 in the presence of VEGF (20 ng mL^−1^) specified after staining with DCFH-DA by flow cytometry (scale bar: 20 μm). **b** Detection of ROS based on fluorescence intensity using prism 8.0 software in HUVE and 4T1 cells after treatments. **c** Flow cytograms of cell apoptosis in HUVE and 4T1 cells induced by PBS, GNPs, VGB3, and GNP–VGB3 in the presence of VEGF (20 ng mL^−1^) using Annexin V/PI staining. **d** The total cell apoptosis in HUVE and 4T1 treated cells were obtained from the sum of early and late apoptosis, which placed at the corner of each panel (in lower-right (Annexin V-FITC+, PI−), and upper-right (Annexin V-FITC+, PI+) quadrants, respectively) and represented in the diagram using prism software. (All data analyzed based on mean ± SEM; One-way ANOVA; n = 3; number sign symbol (#) was used for comparing the treatments with control, and asterisk symbol (*) was used for comparison between treatments, ****P < 0.0001, ***P < 0.001, **P < 0.01, *P < 0.05, or ns: not significant)

Inhibition of VEGF binding to VEGFRs on the endothelial and 4T1 cells results in apoptosis induction [39]. On the other hand, ROS overproduction can activate the apoptotic signaling pathways and cell death [40]. We therefore evaluated the potential of GNP–VGB3 to induce ROS overproduction followed and apoptosis induction in VEGF-induced endothelial and tumor cells. First, the intracellular ROS production was measured in HUVECs and 4T1 cells after treatment with free GNPs, free VGB3, and GNP–VGB3. When treated with concentrations equal to IC_50_ values and in the presence of VEGF (20 ng mL^−1^), the fluorescence intensity of 2-7-dichlorofluorescin diacetate (DCFDA) as ROS production probe in response to GNP–VGB3 treatment were 40.98 and 19.03 in HUVECs and 4T1 cells, respectively, which were significantly higher than that of GNP (16.11 and 8.00, respectively), free peptide (27.28 and 15.69, respectively) and untreated cells (13.8 and 5.24, respectively) (Fig. 5a, b), indicating that neutralization of VEGF receptors led to the overproduction of ROS.

To test GNP–VGB3-mediated induction of apoptosis, we conducted Annexin V and propidium iodide (PI) stainings [41] in VEGF (20 ng mL^−1^)-induced HUVE and 4T1 cells treated with GNP, VGB3 and GNP–VGB3. As indicated in Fig. 5c and d, the percentage of apoptotic cells remarkably increased from 30.98 and 50.83% in GNP- and VGB3-treated cells, respectively, to 74.50% in GNP–VGB3-treated HUVECs. More strikingly, the proportion of apoptotic 4T1 cells were increased in GNP–VGB3-treated group (82.00%) compared to the cells treated by VGB3 (12.35%) and GNP (1.92%) (Fig. 5c, d).

### Inhibition of breast tumor growth in mice

**Fig. 6:**
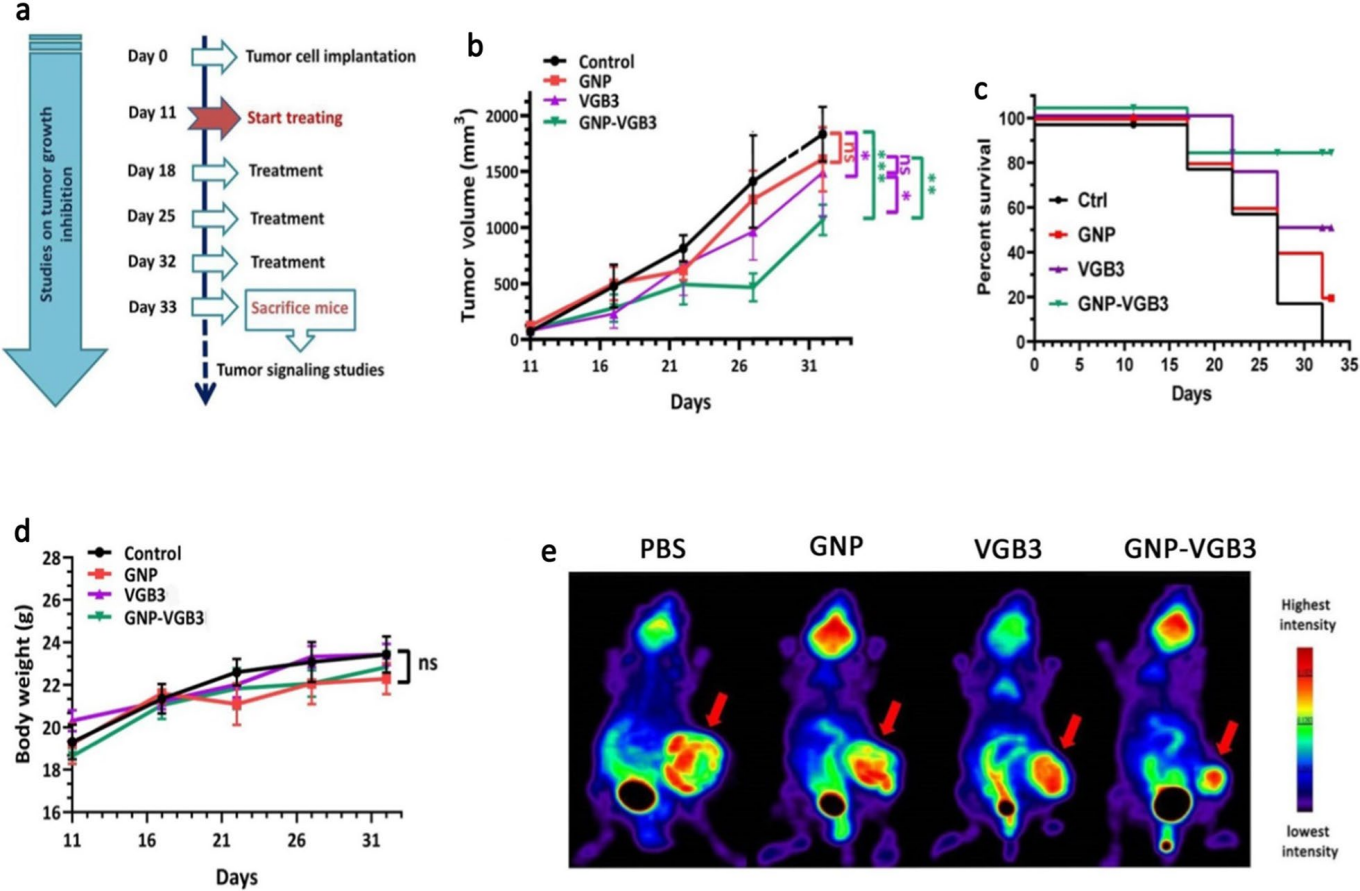
In vivo antitumor activity of PBS, GNP, VGB3, and GNP–VGB3 in murine 4T1 mammary carcinoma tumor model. **a** Schedule for animal experiments. **b** Tumor growth inhibition; lines, mean tumor volume for each group of six animals per group;  error bars signify ± SEM. n = 6; **P < 0.01, ***P < 0.001; two-way ANOVA (**c**) The survival rates of 4T1-bearing mice after treatment were illustrated by Kaplan–Meier curves., and **d** Body weight measurements taken every 5 days until day 32, presented as mean ± SEM. All groups compared with control and analyzed by prism by two-way ANOVA statistical analysis, mean ± SEM and n = 6 (***P < 0.001, **P < 0.01, ns: not significant). **e** [^18^F]-fluorodeoxyglucose (^18^F-FDG) PET imaging of mice treated with GNP, VGB3, GNP–VGB3 or PBS at day 32. Representative PET images are shown with arrows indicating 4T1 mammary carcinoma tumors

To determine whether the superior in vitro potency of GNP–VGB3 over free peptide and GNP is recapitulated in vivo, mice harboring 4T1 mammary carcinoma tumors, which is known as a VEGF-dependent model, were treated with GNP–VGB3, GNP, free peptide or phosphate buffer saline (PBS) as control. When tumor size reached an average volume of ~ 100 mm^3^, different treatment groups intravascularly (i.v.) injected once a week for 3 weeks (Fig. 6a), and during this period, tumor volume, body weight and survival curve were measured. On day 32, the average tumor volume in the GNP–VGB3-treated group (1062 mm^3^) was significantly lower than in PBS (1828 mm^3^), GNP (1605 mm^3^), and free VGB3 (1485 mm^3^). These results indicate that tumor regression occurred in both VGB3 and GNP–VGB3-treated groups, but GNP–VGB3 was significantly more effective. Notably, GNP group showed no tumor growth inhibition (Fig. 6b).

To further assess the in vivo efficacy of treatments, animal survivals was followed up in the treatment groups (n = 6) and the results were compared with PBS-treated controls. The survival curve deduced from Kaplan–Meier analysis until the day 32 after implantation indicated that GNP–VGB3 (one mouse dead; 83.2% survival at day 32) prolonged the survival rate more than VGB3 (two mice dead; 64% survival at the day 32) and GNP (four mice dead; 16% survival at day 32) (Fig. 6c). All members of the control group were lost before day 32. In addition, the body weight of all animals was increased during the treatment period (Fig. 6d), suggesting that the treatments are nontoxic at the dosages used in this work.

To evaluate the effect of treatments on the tumor progression in Balb/c mice, we performed ^18^F-FDG-PET imaging at the end of treatments (day 32). The FDG-PET images of Balb/c mice is presented in Fig. 6e. After treatment for 4 weeks (day 32), the mean uptake values of ^18^F-FDG was more significantly decreased in GNP–VGB3 group than VGB3 group compared within the group that received PBS, whereas the uptake value did not change in GNP group. These results provided strong suggestive evidence that the VGB3-mediated inhibition of tumor progression is improved by gold nanoformulation.

### Suppression of VEGFR-1/-2-mediated signaling in 4T1 mammary carcinoma tumors

Our recent study demonstrated that VGB3/48 h is an effective treatment for metastatic murine 4T1 mammary carcinoma tumors [6] through the inhibition of tumor cells proliferation (decreased Ki-67 expression), angiogenesis (decreased expression of CD31 and CD34), and the induction of apoptosis in tumors (increased TUNEL staining and p53 expression, and decreased Bcl-2 expression) [13]. Results of the current study revealed that weekly treatments attenuate the peptide efficacy, but conjugation to GNP significantly improved its antitumor properties. To explore the molecular mechanisms underlying the superior antitumor effects of GNP–VGB3 against 4T1 mammary carcinoma tumors compared with free peptide, tumors were harvested at the end of the treatment period (day 32 after implantation) and the VEGFR1/R2 signaling pathways were assessed by western blot.

**Fig. 7:**
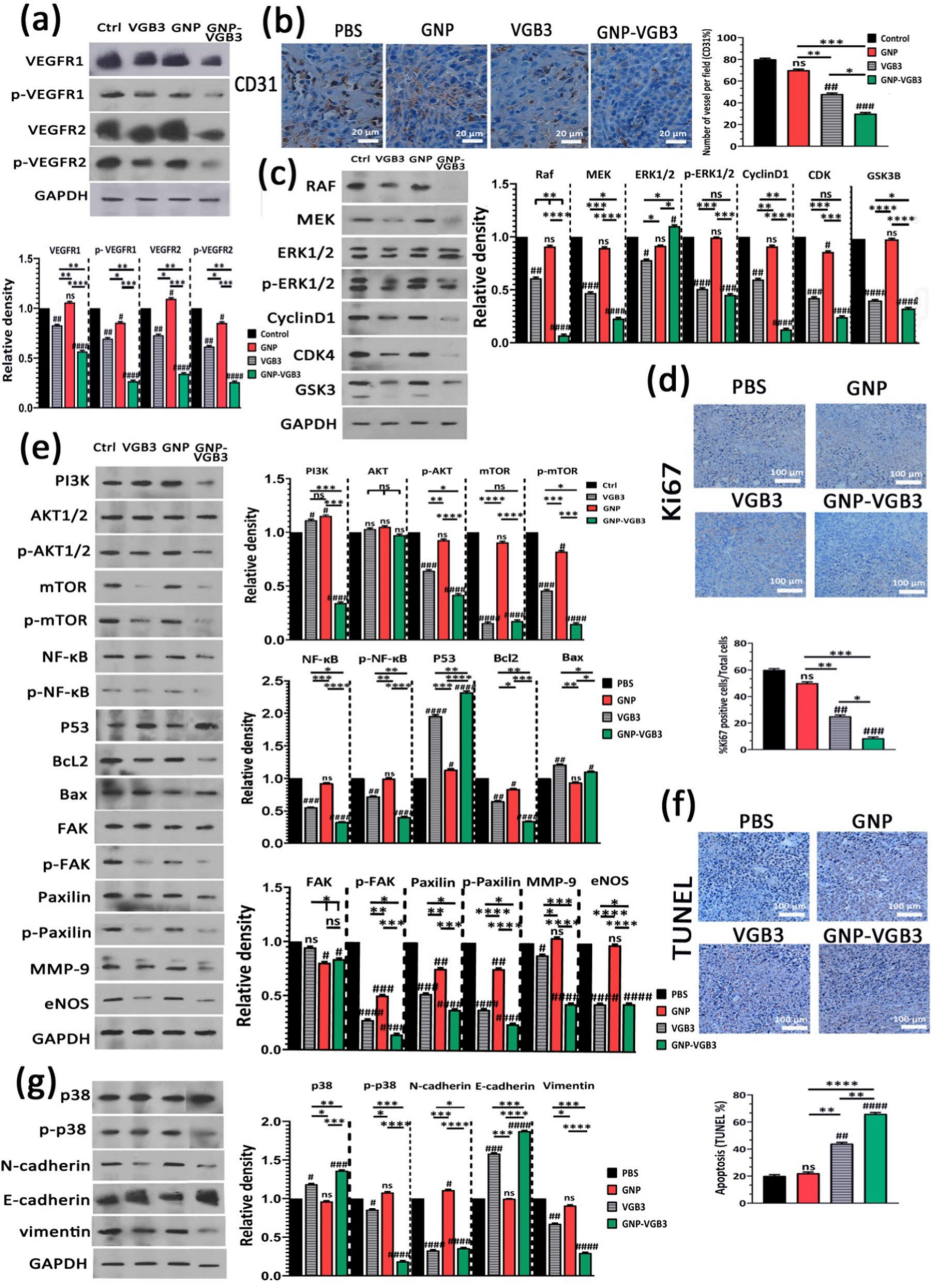
Inhibition of VEGFR1/2-mediated signaling after treatment with PBS, GNP, VGB3 or GNP–VGB3 in 4T1 mammary carcinoma tumor-bearing mice. All of the measurements were done at the end of the treatment period (day 32). **a** Tumor lysates were probed and quantitatively analyzed for levels of total and phosphorylated VEGFR1 and VEGFR2. **b** Representative images and quantitative analysis of CD31 as microvessel formation index (Scale bar = 20 µm). **c** Tumor lysates were probed and quantitatively analyzed with the indicated antibodies. **d** Representative images and quantitative analysis of Ki67 as tumor proliferation index (Scale bar = 100 µm). **e** Tumor lysates were probed and quantitatively analyzed with the indicated antibodies. **f** Representative images of TUNEL and the statistical graph based on the percentage of apoptosis cells (Scale bar = 100 µm). **g** Tumor lysates were probed and quantitatively analyzed with the indicated antibodies. All data were analyzed by prism software (One-way ANOVA method, mean ± SD, n = 3, number sign symbol (#) was used for comparing the treatments with control, and asterisk symbol (*) was used for comparison between treatments ****P < 0.0001, ***P < 0.001, **P < 0.01,*P < 0.1, ns: not significant compared to untreated control)

First, tumor lysates were analyzed for total and phosphorylated VEGFR-1 and VEGFR-2. VEGFR1 and VEGFR2 expressions were abundant in 4T1 tumors, and their expression levels were roughly equivalent in untreated tumors (Fig. 7a). This result, in accordance to our previous investigations [6, 8, 9, 13], confirms that 4T1 model is appropriate for investigation of responses to VEGFR1 and VEGFR2 inhibition. Obviously, VEGFR1 and VEGFR2 expression levels were much more effectively inhibited when weekly treated by GNP–VGB3 (P < 0.0001) than VGB3 (P < 0.01) and GNP compared to controls.

Obviously, blockade of VEGFR1- and especially VEGFR2-mediated signaling attenuates angiogenesis. To assess whether the antitumor effect of GNP–VGB3 is associated with the inhibition of angiogenesis, the microvascular density (MVD) was quantified by immunohistochemical staining of CD31, as an index of angiogenesis. Compared to PBS-treated control group, MVD was significantly reduced in VGB3-treated tumors (P < 0.01) (Fig.  7b); however, the greatest reduction was observed in the GNP–VGB3 group (51% reduction, P < 0.001) compared to controls. In GNP group, there was no significant reduction in MVD (P > 0.9999).

The VEGFR-2 and, to a lesser extent, VEGFR1 has been proved to mediate various cellular signal transduction, including endothelial and tumoral cell survival, proliferation, migration, and induction of permeability [3]. Therefore, the consequences of VEGFR-1/-2 blockade on the constitutive and phosphorylated forms of common downstream proteins were assayed by immunoblotting of the tumor lysates.

The RAS/RAF/MEK/ERK signaling pathway is crucial for the regulation of different cellular processes, including cell proliferation, differentiation and migration. Through cyclin D1 and cyclin-dependent kinases (Cdk)-2/-4, RAS/RAF/MEK/ERK pathway is involved in the regulation of cell cycle progression. We investigated the effects of different treatments on this pathway by measurement of the expression levels of RAF, MEK, CyclinD1, CDK and constitutive and phosphorylated forms of ERK1/2. The results showed that GNP is mostly ineffective on these signaling mediators. Furthermore, GNP–VGB3 resulted in downregulation of RAF, MEK, cyclinD1, CDK-4 and phosphorylated form of ERK1/2 more potently than VGB3, which indicate that the superior antitumor effect of GNP–VGB3 than free peptide is associated with more effective inhibition of proliferation signaling. In agreement with these results, immunohistochemical staining of Ki-67, an index tumor cell proliferation, was decreased by GNP–VGB3 (P < 0.001) more potently than VGB3 (P < 0.01) compared to controls (Fig. 7d). Furthermore, suppression of proliferation signaling supported by decreased expression of glycogen synthase kinase-3 (GSK-3), an inhibitor of cyclin D1, in VGB3- and GNP–VGB3-treated tumors (P < 0.0001) compared to controls (Fig. 7c).

Supporting cell survival and inhibition of apoptosis is another consequence of RAS/RAF/MEK/ERK signaling. Moreover, VEGF promotes cell survival, cancer development as well as metastasis via the PI3K/Akt/mTOR signaling pathway [42]. Accordingly, we sought to further investigate the effects of treatments on the survival, apoptosis and metastasis by analysis of PI3K, AKT, p-AKT, mTOR and p-mTOR, NF-κB, p-NF-κB, P53, Bcl2 and Bax. Compared to controls, the expression level of PI3K increased after treatment with GNP and VGB3 (P < 0.05) but markedly decreased after GNP–VGB3 treatment (P < 0.0001) (Fig. 7e). In agreement with these results, GNP–VGB3 potently reduced p-AKT formation in 4T1 tumors (P < 0.0001), whereas VGB3 was less effective (P < 0.001) and GNP had no effect. mTOR known as promoter of tumor cell migration and invasion [43]. GNP could not change the expression level of mTOR and p-mTOR. In contrast, both VGB3 and GNP–VGB3 treatments drastically suppressed total mTOR expression (P < 0.0001). Furthermore, phosphorylation of mTOR was inhibited more effectively by GNP–VGB3 (P < 0.0001) than by free VGB3 (P < 0.001) compared to PBS-treated tumors. A major target of Akt is the NF-κB pathway [44]. Analysis of tumors revealed highly significant decrease in the expression of NF-κB in VGB3- and GNP–VGB3-treated tumors (P < 0.001 and P < 0.0001, respectively). More importantly, GNP–VGB3 resulted in strong suppression of NF-κB phosphorylation, whereas GNP had no effect and VGB3 group modestly showed NF-κB phosphorylation. Given that activation of NF-κB leads to blockade of apoptosis and promotion of cell proliferation [45], downregulation of NF-κB and p-NF-κB is expected to induce apoptosis in tumors. Accordingly, VGB3-treated tumors present with much higher P53 levels than control 4T1 tumor lysates (P < 0.0001); however, P53 even more increased upon administration of GNP–VGB3 (Fig. 7e). In parallel, GNP–VGB3 treatment resulted in a decrease Bcl2 expression with a concomitant increase in the protein level of Bax (Fig. 7e). These data, consistent with ROS overproduction and annexin V staining in HUVE and 4T1 cells, suggest that GNP–VGB3 enhanced the VGB3-driven inhibition of survival signaling, leading to apoptosis induction in 4T1 mammary carcinoma tumors. To corroborate these results, tumors were analyzed with the TUNEL apoptosis assay. Notably, whereas VGB3 treatment alone had a low effect on the TUNEL-positive cells (P < 0.01), the conjugation of VGB3 to GNP appeared to reinforce the apoptosis induction property of the VEGFR1/2-blocking peptide, as evidenced by the marked increasing of the TUNEL-positive tumor cells in GNP–VGB3-treated tumors (P < 0.0001) (Fig. 7f).

FAK/Paxillin signaling axis, in downstream of PI3K/AKT and MAPK/ERK signaling pathways, is involved in cell adhesion, migration, proliferation, and survival. Analysis of tumor lysates revealed a moderate reduction of total FAK for both VGB3 and GNP–VGB3-treated tumors (P < 0.05) while Paxillin expression was considerably decreased in VGB3 and GNP–VGB3-treated tumors compared to control (P < 0.001 and P < 0.0001, respectively). More strikingly, p-FAK as well as p-paxillin formation were strongly inhibited by all treatments (P < 0.0001) so that GNP–VGB3 and GNP were the most and less effective groups, respectively. These results are indicative of more efficient suppression of cell detachment, as an initial step in the metastatic transformation, by GNP–VGB3 than by other treatments (Fig. 7e).

The process of cell invasion is a combination of cell migration with concurrent degradation of the surrounding extracellular matrix (ECM) by matrix metalloproteases [46]. Decreased expression of MMP-9 has been observed in 4T1 mammary carcinoma tumors treated with VEGF blockading peptides [9]. Importantly, GNP–VGB3 potently inhibited MMP-9 expression in tumor tissue (P < 0.0001), whereas free peptide was moderately effective (P < 0.05) and GNP had no effect (Fig. 7e).

Inhibition of VEGFR2 phosphorylation was shown to inhibit metastasis and cancer progression via eNOS/Akt signaling [47], underlined by the fact that endothelial nitric oxide synthase (eNOS) induces nitric oxide (NO) production, which plays important role in vascular protection, focal adhesion formation and cell migration. Our results indicated that GNP-treatment has no effect on eNOS expression in tumor tissues, whereas both VGB3 and GNP–VGB3 treatments markedly suppressed eNOS expression compared to controls (P < 0.0001) (Fig. 7e).

p38 MAP kinase has been implicated in a variety of cellular processes, including cell proliferation, cell differentiation, apoptosis, cell migration, and invasion [48, 49]. The total expressions of p38 MAPK were unaffected by GNP treatment but increased by VGB3 (P<0.05) and GNP–VGB3 (P < 0.001) compared to controls. More strikingly, p38 MAP kinase activation, i.e. p-p38 MAP kinase formation, strongly suppressed by GNP–VGB3 (P < 0.0001) but not by the other treatments compared to controls (Fig. 7g).

Epithelial–mesenchymal transition (EMT) promotes metastasis by enhancing mobility, invasion, and resistance to apoptotic stimuli [50]. Importantly, EMT is characterized by decreased expression of cell adhesion molecules such as E-cadherin and increased expression of vimentin and N-cadherin. We therefore compared the potential of treatments to affect the expression of E-cadherin, vimentin and N-cadherin. GNP–VGB3 treatment was more effective than VGB3 in decreasing the expression of vimentin and N-cadherin as well as in increasing the expression of E-cadherin in 4T1 tumors (P < 0.0001), whereas the expression levels of both proteins in GNP-treated tumors were comparable with controls (Fig. 7g). These results indicate that GNP–VGB3 attenuated EMT in 4T1-bearing Balb/c mice.

**Fig. 8:**
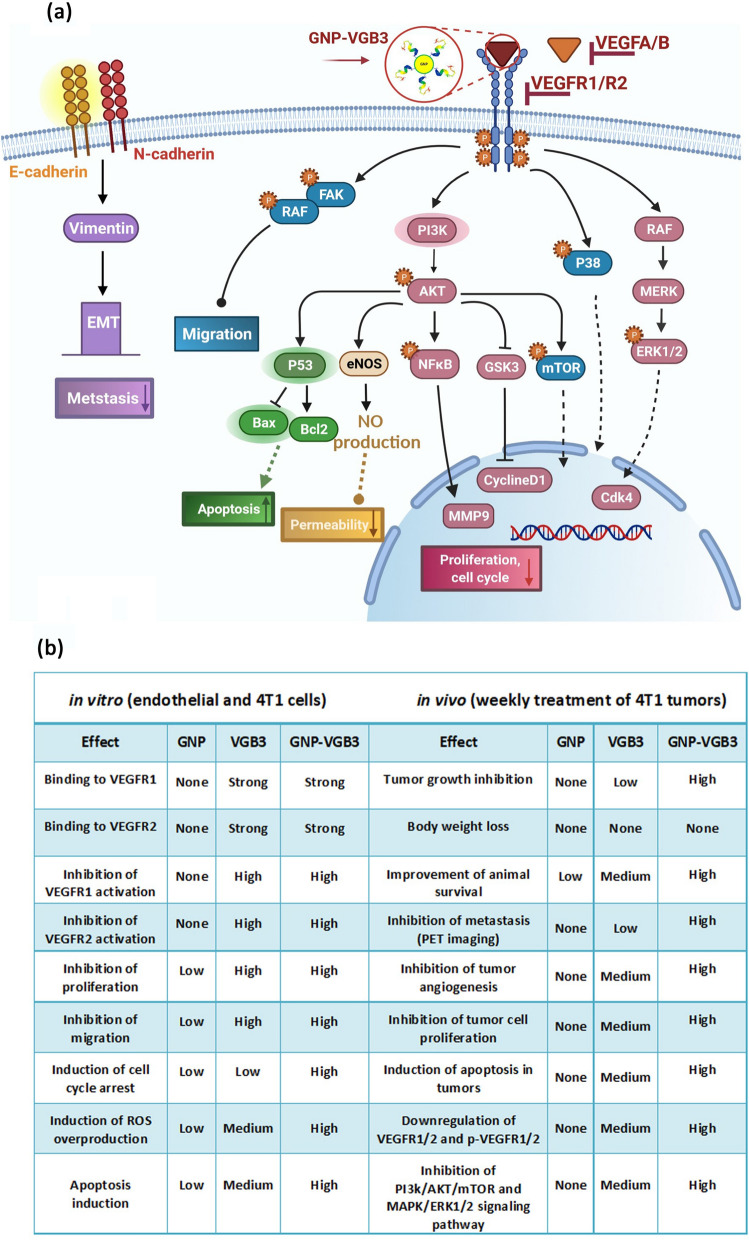
Signaling transduction and biological processes mediated by inhibition of VEGFR1 and VEGFR2 using GNP–VGB3. **a** Schematic illustration of the effect of GNP–VGB3 on the downstream signaling pathways of VEGFR1/2. Binding of GNP–VGB3 to VEGFR1/R2 inhibits the activity of receptors that were activated by VEGFA/B and the downstream signaling pathways is subsequently prevented. VGB3 after conjugation to GNPs suppresses the signaling pathways in via preventing cell proliferation, migration, apoptosis, permeability and metastasis. **b**  The comparison of in vitro and in vivo effects exerted free GNP, free VGB3 peptide and GNP-VGB3

Although most of known responses to VEGFA are mediated by VEGFR2, endothelial cell functions can be stimulated by VEGFR1 especially through PI3K/Akt pathway [51]. In agreement with the in vitro results, analysis of signaling pathways in tumor tissues showed that even greater inhibitory effects than those resulted from VEGFR1/VEGFR2 blockading peptide can be obtained from its combination with the positive effects of GNP. Figure 8 represents the wide ranges of signaling mediators targeted by GNP–VGB3.

### MicroCT imaging

**Fig. 9:**
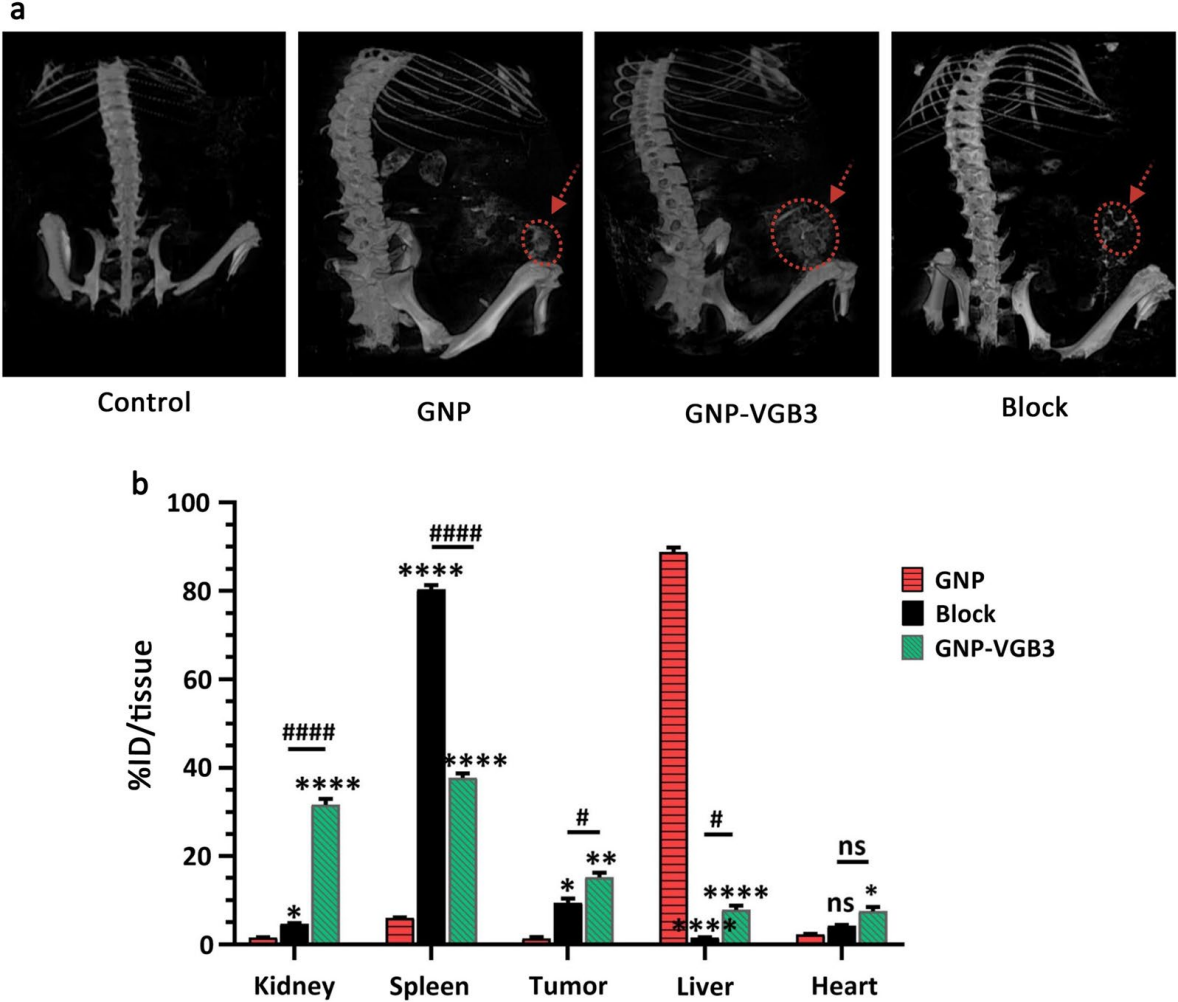
Biodistribution study by MicroCT imaging and ICP MS analysis. **a** Representative 3D-reconstructed whole-body CT images of mice bearing 4T1 tumors at 3 h following intravenous (i.v.) injection of GNP, GNP–VGB3, block and PBS (untreated control). The red circles and arrows indicate tumor locations. **b** The Au concentrations in major organs, including kidney, spleen, tumor, liver and heart, which received GNP, GNP–VGB3 and block samples quantified after 24 h using inductively coupled plasma mass spectrometry (ICP-MS) analysis. The quantified results were defined based on the percentage of injection dose per tissue (%ID/tissue) and analyzed statistically by two-way ANOVA method (mean ± SEM, and n = 3, ****P < 0.0001, **P < 0.01, *P < 0.05, or ns: not significant). (Asterisk symbol (*) was used for comparison between GNP with GNP–VGB3 and block and number sign symbol (#) was used for comparing GNP–VGB3 and block)

To identify the accumulation of nanoparticles in the tumors, we examined mice treated with the GNP, VGB3 and GNP-VGB3  using MicroCT imaging. Initially, one group of 4T1 tumor-bearing Balb/c mice (n = 3) were intravenously injected with free GNPs. After injection for 3 h, the strongest CT signals were observed in kidneys (Fig. 9a). In addition, CT signals were observed in tumors and liver (Fig. 9a).  However, the signals in the tumor regions were augmented in mice treated with peptide bound GNP (GNP–VGB3) (Fig. 9a). Furthermore, target specificity of GNP–VGB3 elicited by a blocking experiment. As shown in Fig. 9a, accumulations were suppressed effectively by coadministration of competing free VGB3 peptide. These observations suggest that GNP–VGB3 can specifically target mammary carcinoma tumors.

### Biodistribution study of nanoparticles

Quantitative biodistribution analysis was performed by examining the gold content (% ID/tissue). To this end, the mice were sacrificed, a series of dissected organs (kidney, spleen, liver and heart) and tumor tissues of mice (n = 3) were freshly collected 24 h post-injection, and inductively coupled plasma mass spectrometry (ICP-MS) measurements were immediately taken. As shown in Fig. 9b, a very high amount of gold was found in liver (88.8% ID/tissue) and spleen (80.3% ID/tissue) for GNP and blocking groups (n = 3), respectively, suggesting that these nanoparticles are cleared mainly through the reticuloendothelial system. In these groups, the tumor accumulations were 1.3 and 8% ID/tissue, respectively. The tumor accumulation of GNP–VGB3, however, increased to 15.2% ID/tissue, which was significantly more than those of GNP (1.4%) and blocking (9.4%). Importantly, the highest accumulation of GNP–VGB3 was observed in kidney. The observation that free GNP is accumulated mostly in the liver was also reported in previous investigations [52, 53]. Nanosystems with renal clearance are more desirable than those cleared through the reticuloendothelial system [54]. Thus, higher accumulation of GNP–VGB3 kidney may induce less damage than free GNP in the normal tissues. These data suggest that the tumor accumulation of GNP–VGB3 is more efficient than free GNP. In addition, a decreased amount of GNP–VGB3 in the presence of free peptide (block) further confirms its specific binding to tumors.

## Conclusion

Dual inhibition of VEGFR1 and VEGFR2 leads to antiangiogenic and antitumor outcomes beyond the inhibition of VEGFR2 alone. It has been shown earlier that VGB3 is a dual VEGFR1/R2 blockading peptide that inhibit growth and metastasis of breast tumors through abrogation of angiogenesis as well as inhibition of proliferation and migration, and induction of apoptosis in 4T1 tumor cells. The current research revealed that VGB3-decorated gold nanoparticles are more suited for this purpose. Although free GNPs could not produce inhibitory effects, our in vitro and in vivo results demonstrated that GNPs potentiate the anticancer properties of VGB3. Based on mechanism-based analyses of tumor tissues, the superior anticancer properties of GNP–VGB3 over free peptide is due to retaining the VEGFR1/R2-binding property, followed by suppression of signaling pathways of proliferation, migration and metastasis in tumor-bearing animals. These data open a new window to understand how antiangiogenic, antitumor and antimetastatic properties arising from the dual blockade of VEGFR1 and VEGFR2 can be potentiated by the positive therapeutic effects of gold nanoparticles. We also believe that GNP–VGB3 is a promising candidate for clinical translation. While the focus of current investigation for GNP-peptide conjugate was increasing the therapeutic efficacy of the antiangiogenic peptide, the results of CT and organ distribution indicated that this nanosystem can also be useful in CT imaging of tumors. This may be of particular interest since VGB3 binds to VEGFR1 and VEGFR2, which are highly expressed in variety of tumors.

## Supplementary Information

**Additional file 1: Figure S1.** (a) Peptide was purified as 90% by high-performance liquid chromatography (HPLC). (b) The molecular structure of the peptide and the disulfide bond formation was confirmed by electrospray ionization-mass spectrometry (ESI-MS). **Figure S2.** The verification of the size and the shape of synthesized GNP–VGB3 by FESEM. **Figure S3.** Ability of GNP, VGB3 and GNP–VGB3 in binding to VEGFR1, and VEGFR2 on HUVECs and their dose dependent manner. **Figure S4.** Ability of GNP, VGB3 and GNP–VGB3 in binding to p-VEGFR1, and p-VEGFR2 on HUVECs and their dose dependent manner.

## Data Availability

The data set that support the findings of this study are available from the corresponding author on reasonable request.

## Abbreviations

GNPs: Gold nanoparticles
HUVECs: Human umbilical vein endothelial cells
VEGF: Vascular endothelial growth factor
VEGFR: Vascular endothelial growth factor receptor
TKIs: Tyrosine kinase inhibitors
MUA: 11-Mercapto undecanoic acid
MU: Mercapto undecanol
MES: 2-(*N*-Morpholino) ethanesulfonic acid
EDC: *N*-(3-Dimethylaminopropyl)-*N*′-ethylcarbodiimide hydrochloride
BSA: Bovine serum albumin
HPLC: High-performance liquid chromatography
ESI-MS: Electrospray ionization-mass spectrometry
SPR: Surface plasmon resonance
DLS: Dynamic light scattering
FAAS: Flame atomic absorption spectrometry
ICP-MS: Inductively coupled plasma-mass spectrometry
FT-IR: Fourier transform infrared spectroscopy
AFM: Atomic force microscopy
FESEM: Field emission scanning electron microscopy
TEM: Transmission electron microscopy
EDS: Energy dispersive X-ray spectroscopy
RPMI-1640: Roswell Park Memorial Institute media
FITC: Fluorescein isothiocyanate
DAPI: Dark position. 4′,6-diamidino-2-phenylindole
PE: Phycoerythrin
PI: Propidium iodide
ROS: Reactive Oxygen Species
DCF-DA: 2ʹ, 7ʹ-Dichlorodihydrofluorescein diacetate
IACUC: Institutional Animal Care and Use Committee
PET: Position emission tomography
IHC: Immunohistochemical
DAB: 3,3-Diaminobenzidine
PVDF: Polyvinyl difluoride
HRP: Horseradish peroxidase
ECs: Endothelial cells
PBS: Phosphate buffer saline
i.v.: Intravascularly
GSK-3: Glycogen synthase kinase-3
