# Supplementary material for: Conjugation of VEGFR1/R2-targeting peptide with gold nanoparticles to enhance antiangiogenic and antitumoral activity
Source: J Nanobiotechnology. 2022 Jan 4;20:7. doi: 10.1186/s12951-021-01198-4 (PMC8725421; doi:10.1186/s12951-021-01198-4)
Supplement: Supplementary file 1 — Additional file 1: Figure S1. (a) Peptide was purified as 90% by high-performance liquid chromatography (HPLC). (b) The molecular structure of the peptide and the disulfide bond formation was confirmed by electrospray ionization-mass spectrometry (ESI-MS). Figure S2. The verification of the size and the shape of synthesized GNP–VGB3 by FESEM. Figure S3. Ability of GNP, VGB3 and GNP–VGB3 in binding to VEGFR1, and VEGFR2 on HUVECs and their dose dependent manner. Figure S4. Ability of GNP, VGB3 and GNP–VGB3 in binding to p-VEGFR1, and p-VEGFR2 on HUVECs and their dose dependent manner. [file 12951_2021_1198_MOESM1_ESM.docx]

Additional Material

| **Conjugation of VEGFR1/R2-targeting peptide with gold nanoparticles to enhance antiangiogenic and antitumoral activity** |
| --- |
| Pegah Zanjanchi^1^, S. Mohsen Asghari^2^ *, Hassan Mohabatkar^1^ *, Mostafa Shourian^3^  ^1^Department of Nanobiotechnology, Faculty of Biology science and Technology, Isfahan University, Isfahan, 8174673441, Iran  ^2^Institute of Biochemistry and Biophysics (IBB), University of Tehran, Tehran, 1417614411, Iran  ^3^Department of Biochemistry, Faculty of Sciences, University of Guilan, Rasht, 4199613776, Iran |


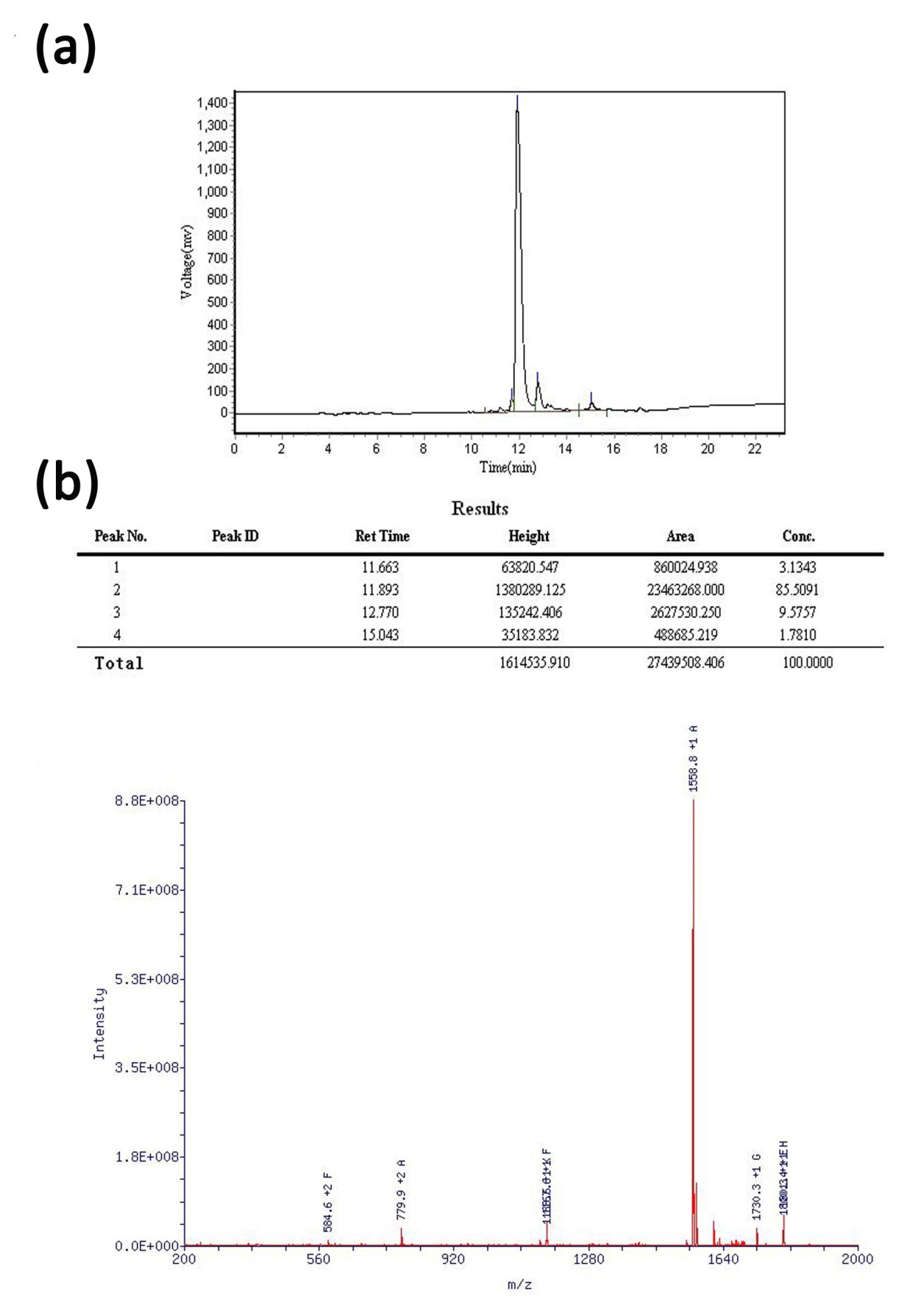


Fig. S1 (a) Peptide was purified as 90 % by high-performance liquid chromatography (HPLC). (b) The molecular structure of the peptide and the disulfide bond formation was confirmed by electrospray ionization - mass spectrometry (ESI-MS).

Fig. S2 The verification of the size and the shape of synthesized GNP-VGB3 by FESEM. FESEM images of GNP-VGB3 with 50.00 KX magnifications and the scale bar of 200 nm. The average diameter of GNP-VGB3 in the image is around 24.6 nm.


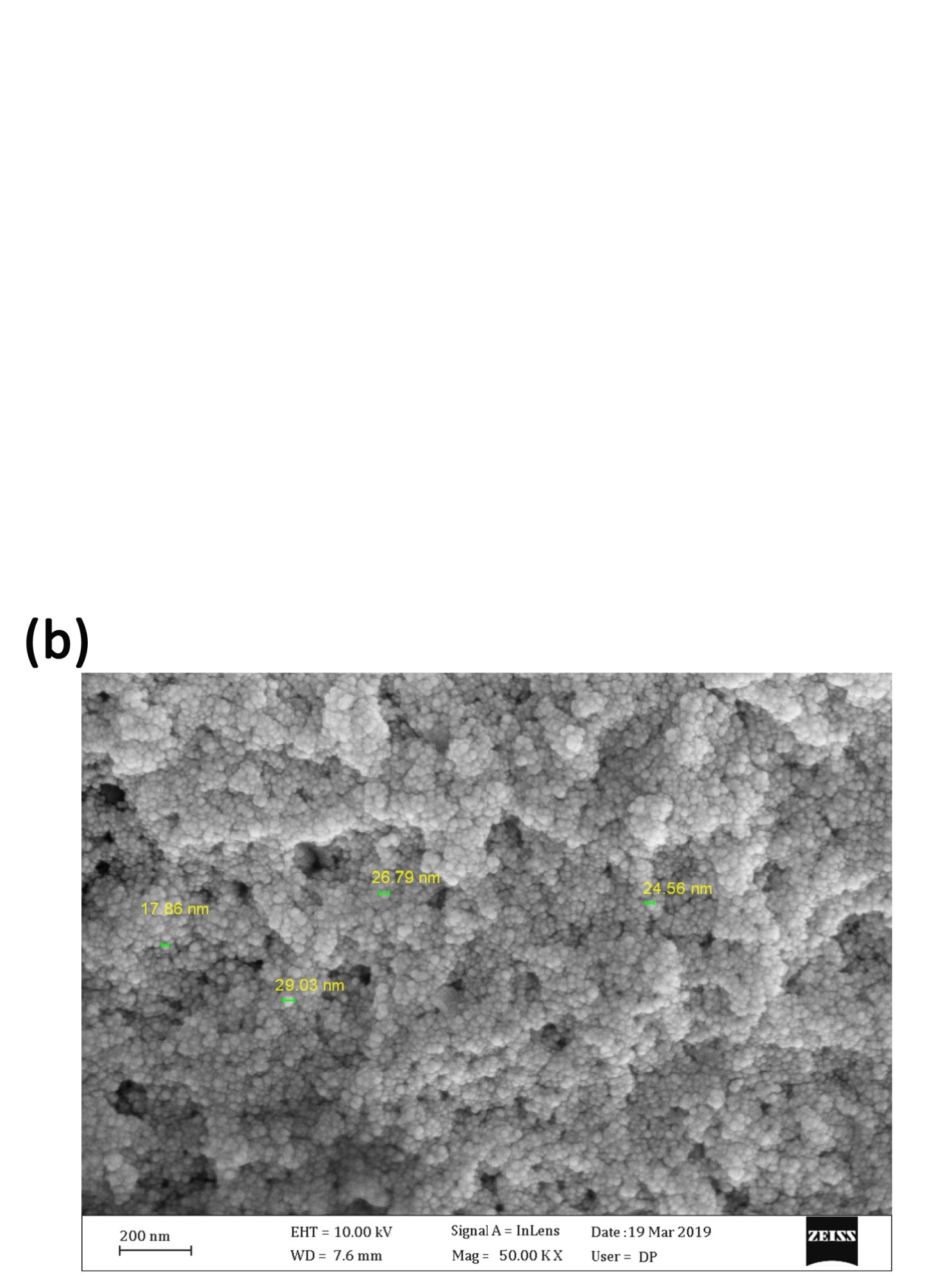


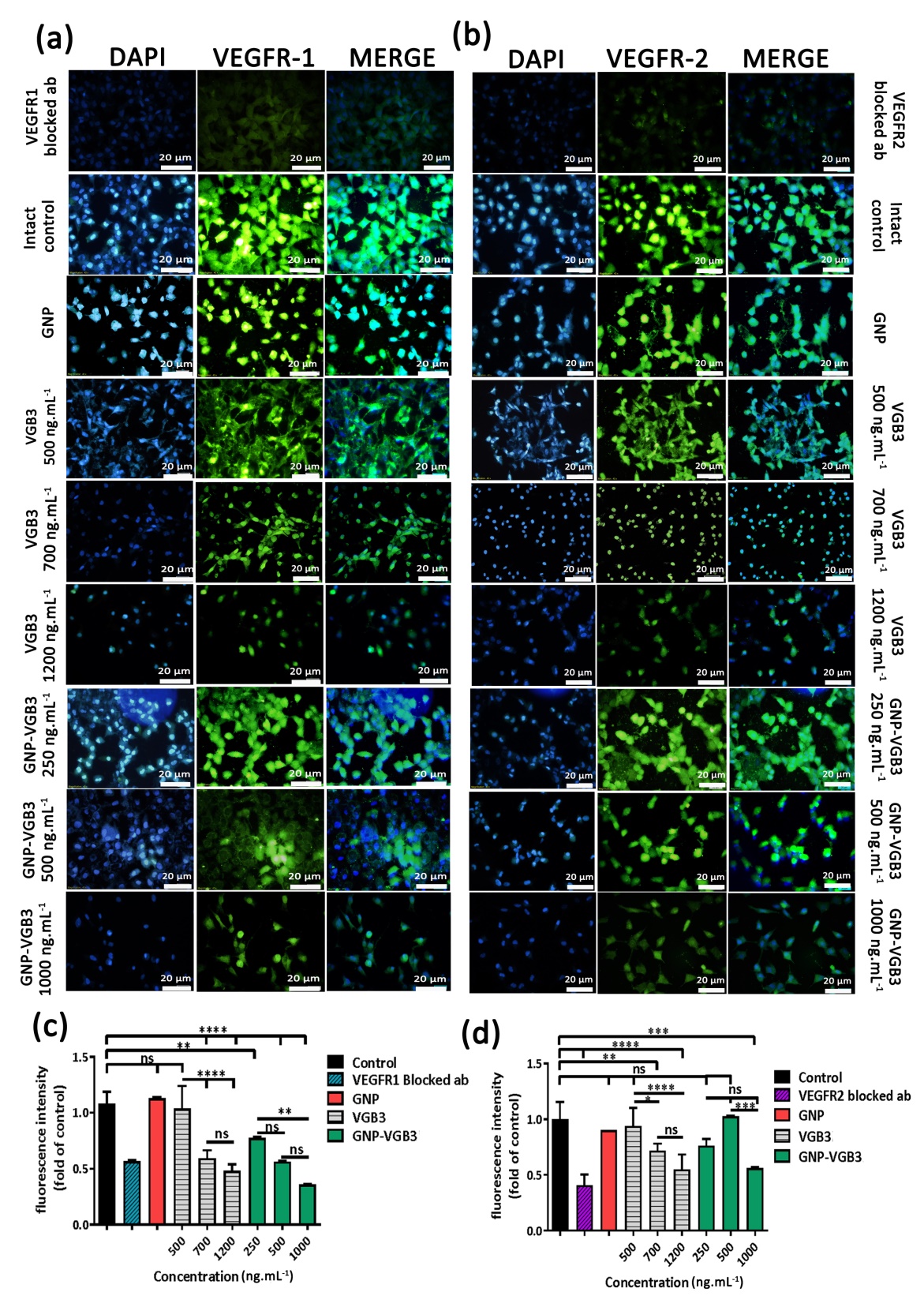


Fig. S3 Ability of GNP, VGB3 and GNP-VGB3 in binding to VEGFR1, and VEGFR2 on HUVECs. Immunoflourescence images of HUVECs treated with GNP, VGB3 and GNP-VGB3 involved in the binding inhibition of (a) anti-VEGFR1 and (b) anti-VEGFR2 both conjugated to FITC-secondary anti-mouse antibody (green) to bind to VEGFR1 and VEGFR2, respectively. HUVECs were treated with different treatments with various concentrations such as GNP, VGB3 (500-1200 ng.mL^-1^), GNP-VGB3 (250-1000 ng.mL^-1^) in compared with untreated control and blocked antibodies of VEGFR1, and VEGFR2. (c) and (d) statistical analysis were performed by prism software 8; Oneway ANOVA and all data displayed mean ± SEM (n=3). The scale bar of all images was 20 μm.


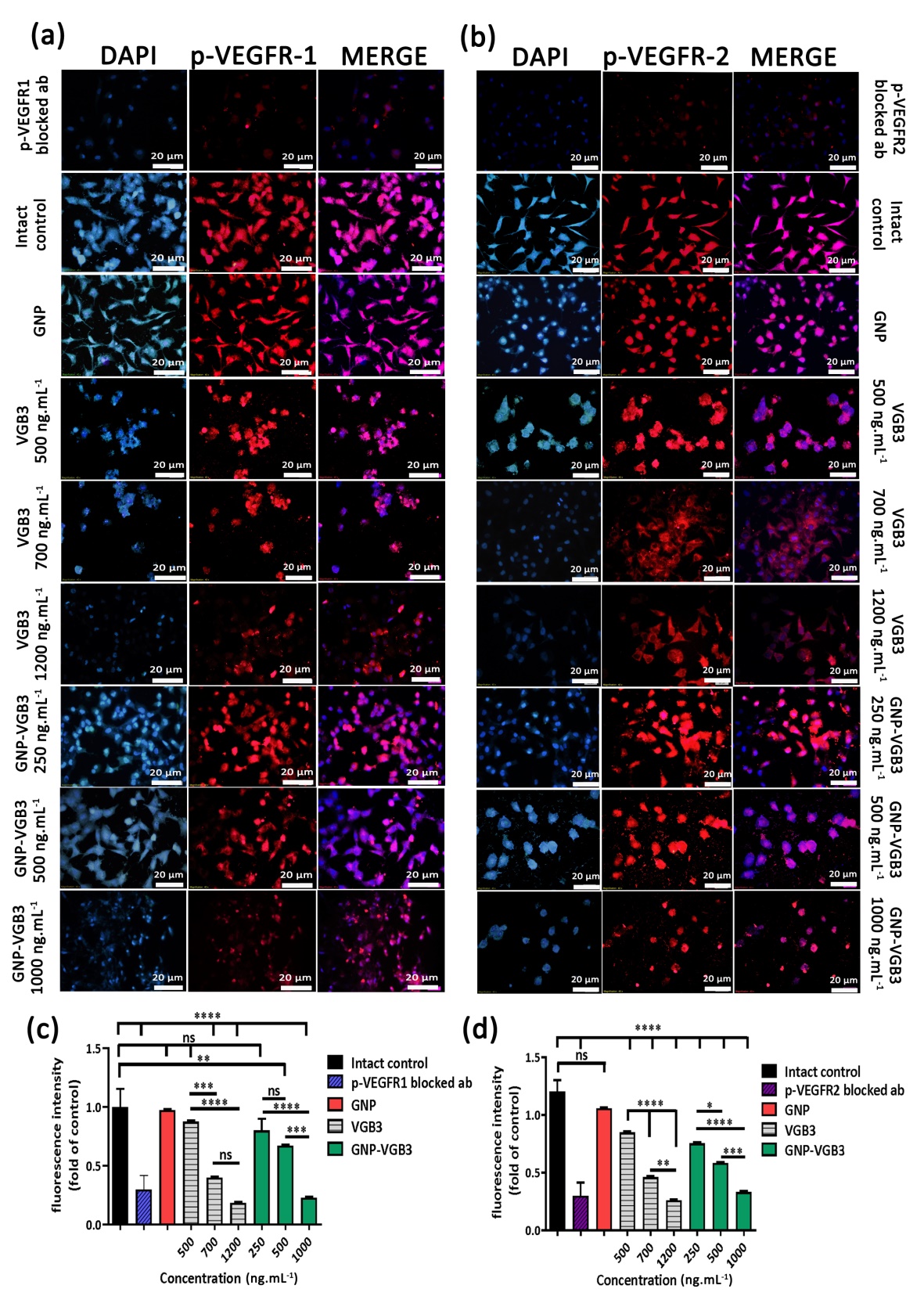


Fig. S4 Ability of GNP, VGB3 and GNP-VGB3 in binding to p-VEGFR1, and p-VEGFR2 on HUVECs. Immunoflourescence images obtained from the inhibition of (a) p-anti-VEGFR1 and (b) p-anti-VEGFR2 together stained with PE-secondary anti-mouse antibody (red) to bind to p-VEGFR1 and p-VEGFR2, respectively in HUVECs treated with GNP, VGB3 and GNP-VGB3. HUVECs were treated with different treatments with various concentrations such as GNP, VGB3 (500-1200 ng.mL^-1^), GNP-VGB3 (250-1000 ng.mL^-1^) in compared with untreated control and blocked antibodies of p-VEGFR1, and p-VEGFR2. (c) and (d) statistical analysis were performed by prism software 8; One Way ANOVA and all data displayed mean ± SEM (n=3). The scale bar of all images was 20 μm.
